# Unique composition and evolutionary histories of large low velocity provinces

**DOI:** 10.1038/s41598-025-88931-3

**Published:** 2025-02-06

**Authors:** James Panton, J. Huw Davies, Paula Koelemeijer, Robert Myhill, Jeroen Ritsema

**Affiliations:** 1 School of Earth and Environmental Sciences, Cardiff University, Park Place, Cardiff, Wales CF10 3AT UK; 2 Department of Earth Sciences, University of Oxford, South Parks Road, Oxford, OX1 3AN England, UK; 3 School of Earth Sciences, University of Bristol, Queens Road, Bristol, BS8 1RJ England, UK; 4 Department of Earth and Environmental Sciences, University of Michigan, 1100 North University Avenue, Ann Arbor, MI 40819 USA

**Keywords:** Geochemistry, Geodynamics, Tectonics

## Abstract

The two “large low velocity provinces” (LLVPs) are broad, low seismic wave speed anomalies in Earth’s lower mantle beneath Africa and the Pacific Ocean. Recent research suggests they contain relatively dense subducted oceanic crust (SOC), but the relative concentration of this recycled material within them is an open question. Using simulations of 3-D global mantle circulation over the past 1 Gyr, we find that two antipodal LLVPs develop naturally as a consequence of Earth’s recent subduction history and the gravitational settling and stirring of SOC. Shear-wave velocity reductions in the two LLVPs are similar due to the dominating influence of temperature over composition. However, the formation histories are distinct. Circum-Pacific subduction of oceanic lithosphere has continuously replenished the Pacific LLVP with relatively young SOC since 300 Ma, while the African LLVP comprises older, well-mixed material. Our models suggest the Pacific LLVP stores up to 53% more SOC produced in the last 1.2 Gyr than the African LLVP, potentially making the Pacific domain denser and less buoyant.

## Introduction

The large low velocity provinces (LLVPs) are broad, spherical harmonic degree 2, seismic wave speed anomalies in the lower mantle beneath the Pacific and Africa^1^. Their composition, formation, and longevity have been the focus of many geodynamic and seismological investigations^2–5^, as the nature and evolution of these structures provide important insights into the deep and shallow processes that have shaped our planet. Tomographic models consistently find wave speed reductions of 1% to 3% for both the African and Pacific LLVPs^6^, with typically a stronger reduction for the shear-wave velocity than compressional-wave velocity. Together, the two structures cover about 25–30% of the core surface^1^, but the African LLVP may reach up to 550 km higher above the core-mantle boundary (CMB) than its Pacific counterpart^7,8^. Deep rooted mantle plumes are often associated with the interiors and edges of the LLVPs^9^, and the structures themselves are thought to migrate laterally over time^10^. In addition, there may be lateral isotopic variations within each LLVP^11^ as well as depth variations in bulk composition^12^. Following decades of research, the prevailing idea is now that the LLVPs represent anomalously hot regions^13^ with a different bulk chemistry than the ambient mantle and that their locations and geometries are strongly influenced by Earth’s subduction history^14,15^.

There are two end-member views on the formation of thermo-chemical LLVPs. One is that the LLVPs may be enriched in iron-rich, primitive crust^12^ or other primordial material^16,17^. Primordial enrichment may be the result of upside down differentiation^18^ or the moon-forming impact of the proto-Earth with Theia^19^. In either case, LLVPs enriched with a primordial component must be denser and/or more viscous than the surrounding material to resist entrainment into the convecting mantle^20^. Alternatively, and the point of view we take in this study, LLVPs may be areas of the lower mantle that are enriched in subducted oceanic crust (SOC)^21,22^, replenished continuously by subduction^23,24^. The model of LLVPs being enriched in SOC is attractive as seismic tomography informs us that subducted slabs enter the lower mantle^25^, while the geochemical analysis of lavas sourced from deep-rooted plumes typically associated with the LLVPs^26^, show them to contain a significant component of SOC^27–29^.

Previous mantle circulation modeling has demonstrated that the locations at which slabs enter the lower mantle cause primordial thermochemical piles to be pushed together and subsequently split apart again^10,14^ with the evolving overlying pattern of subduction zones. This leads to mobile thermochemical structures at the base of the mantle and indicates a strong relationship between the present day LLVPs and recycling of SOC^30^. As a result of Earth’s reconstructed plate locations, large (degree 2) structures dominate the lowermost mantle for the majority of the last 1 Gyr^15^, indicating that LLVP-like features may have been present for much of Earth’s recent history.

While there have been numerous studies into the formation of LLVPs from a dense primordial layer, e.g.^30–32^, studies of the contribution of SOC to LLVP formation have largely been limited to 2D geometry^23,33,34^, but have been able to illustrate the major controls. Christensen and Hofmann^33^ were the first authors to explicitly attempt to simulate the subduction of oceanic crust into the mantle, finding that SOC could segregate to the lowermost mantle for extended periods of time given a high enough excess density. Work by Li and McNamara^35^ highlighted that SOC near the CMB could readily be entrained by strong plumes, casting doubt over the role that SOC might play in LLVP formation. However, later work by Mulyukova et al.^23^ showed that continuing subduction could replenish the SOC budget of the lower mantle, leading to the idea that thermochemical LLVPs may be compositionally heterogeneous, rather than overwhelmingly dominated by a single composition. Regarding the shape of the LLVPs, several modelling studies indicate that the intrinsic (chemical) density of SOC strongly controls the lateral extent of thermochemical LLVPs, and that the relative viscosity between the LLVPs and the ambient mantle influences their morphology^21,35^. Two-dimensional simulations also show that changing subduction patterns affect the distribution of LLVP material in the lowermost mantle^34^, with accumulations being redistributed around the CMB by incoming slabs. With this previous work in mind, a 3-D approach is the next necessary step to better understand the effect of Earth-like subduction patterns on the formation of LLVPs from SOC.

To investigate in 3-D the transport and accumulation of SOC in the mantle we use the mantle circulation code TERRA^36,37^ (“Dynamic simulations”). A 3-D approach allows us to include Earth-like plate subduction history and perform geographically referenced comparisons between simulations and observations. We estimate the shapes and locations of the LLVPs, how LLVPs develop over time, and map differences in their bulk composition (i.e., SOC enrichment) and age (i.e, most recent melting of SOC) to gain understanding of their long-term evolution.

## Numerical simulations of LLVPs

We solve the governing equations of mantle convection under the Boussinesq approximation, with isothermal boundary conditions and a depth and temperature-dependent viscosity (“Dynamic simulations”). Kinematic reconstructions of plate motions^38,39^ are used to constrain surface plate velocities and therefore the paleo-locations of subduction zones and spreading ridges over the past 1 Gyr. This long simulation time captures the cycle of oceanic crust segregation from the subducted lithosphere, descent through the mantle, accumulation of SOC at the CMB, and entrainment and replenishment^23,24^. The plate reconstructions of Müller et al.^38^ have been constructed to fit hot spot age tracks, optimise trench migration behaviour and minimise net lithospheric rotation. The resulting model gives absolute plate motions in a mantle reference frame, making it ideal for our use case. The precursor plate model by Merdith et al.^39^ is set up in a palaeomagnetic reference frame, which ultimately leads to increasingly larger differences between the two models going back in time.

Oceanic crust is generated via a melting process^40–42^ at mid-ocean ridges, with the material recycled into the mantle in plate convergence zones (“Particles”). The flux of oceanic crust into the mantle is therefore not imposed, instead it is a function of the oceanic crust production rate and the plate velocities. Bulk composition (*C*) is parameterised as a scalar which ordinarily varies between 0 and 1, with three principal lithologies prescribed in our model: harzburgite ($C = 0.0$), lherzolite ($C = 0.2$), and basalt ($C = 1.0$). For simplicity we use this terminology when referring to high pressure assemblages of these lithologies. The temperature and composition fields are converted to seismic velocities (Methods 6.3) and adjusted for the tomographic resolution to facilitate a comparison with S40RTS^43^ (Methods 6.4).

**Fig. 1:**
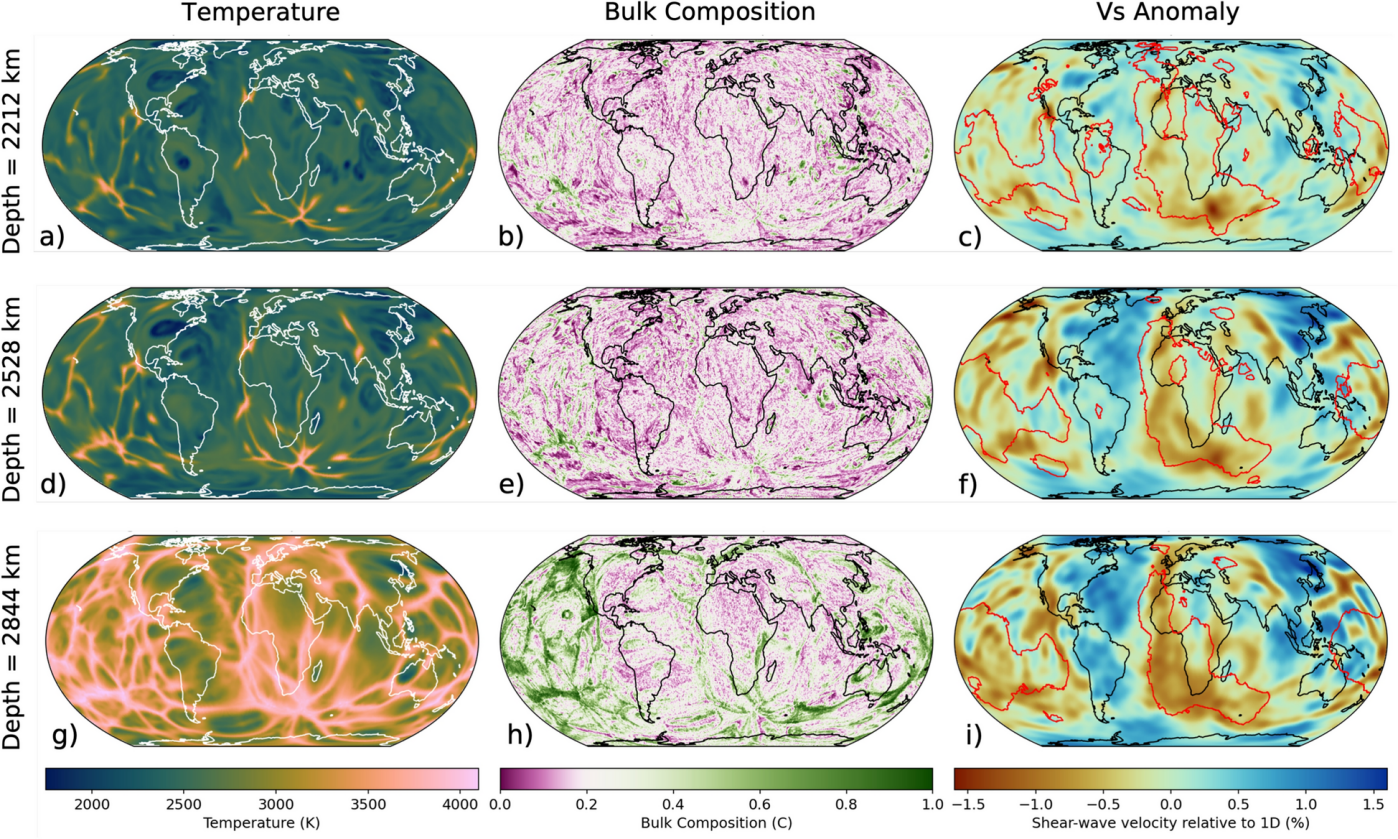
Present day mantle structure predicted by simulation RCY showing temperature (**a**, **d**, **g**), bulk composition (**b**, **e**, **h**), and converted shear-wave speed filtered using the resolution of seismic tomography model S40RTS^43^ (**c**, **f**, **i**) at 2212 km (top tow), 2528 km (middle row) and 2845 km (bottom row) depth. The colour scale for bulk composition is centred on $C = 0.2$, which is the average composition for the mantle, with purple colours indicating depleted material and green colours indicating enriched material. The seismic velocities are obtained by assuming that the bulk composition *C* of the tracer fields represents a mechanical mixture of the 3 principal lithologies (Methods 6.3). Red lines plotted on top of shear-wave velocity maps outline the margins of the LLVPs in a“vote map” of 18 shear wave tomography models^51,52^, indicating where at least 5 models are in agreement.

We focus the discussion on the results of reference simulation RCY (Table 1). Simulation RCY features a radial viscosity profile of three layers (Supplementary Figure S1) with lateral variations in viscosity determined by temperature variations (“Dynamic simulations”). RCY predicts present-day accumulations of SOC in the lower mantle under the Pacific and Southern Ocean (Fig. 1h) and elevated temperatures in the lower mantle beneath the Pacific and southern Africa (Fig. 1g), where plumes are preferentially formed (Fig. 1a,d).

**Table 1:** Table of model parameters, showing the buoyancy number, viscosity profile and CMB temperature used in each simulation. Note that most simulations are incompressible and an adiabatic contribution must be added to compare to Earth. Viscosity profiles are plotted in Supplementary Figure S1.

Name	Buoyancy number of SOC	Viscosity profile	$\mathbf {T_{CMB}}$ (K)	Primordial layer thickness (km)	Plate model
RCY	0.66	visc1	3000	–	Müller et al. (2022)
B = 0.44	0.44	visc1	3000	–	Müller et al. (2022)
B = 0.22	0.22	visc1	3000	–	Müller et al. (2022)
visc2	0.66	visc2	3000	–	Müller et al. (2022)
visc3	0.66	visc3	3000	–	Müller et al. (2022)
CMB2800	0.66	visc2	2800	–	Müller et al. (2022)
CMB2600	0.66	visc2	2600	–	Müller et al. (2022)
PRM	0.66	visc1	3000	150	Müller et al. (2022)
COMP	0.66	visc1	4000	–	Müller et al. (2022)
MER	0.66	visc1	3000	–	Merdith et al. (2021)

The low shear-wave velocity anomalies ($\delta V_{s}$) beneath Africa and the Pacific are in similar locations and have similar shapes as the LLVPs in S40RTS (Fig. 1c, f, i, Supplementary Figure S2). This is also evident from the strong positive correlation between the $\delta V_{s}$ structure in RCY and S40RTS throughout the lower mantle up to spherical harmonic degree 4 (Supplementary Figure S3). Henceforth, we refer to these low $\delta V_{s}$ structures as the simulated large low velocity provinces, or s-LLVPs, but we note that the LLVPs in S40RTS and the s-LLVPs in RCY are not exactly similar. The s-LLVPs in RCY are broader (Supplementary Figure S4), the Pacific s-LLVP is more spatially discontinuous (Fig. 1c, f, i), and the s-LLVP beneath Africa is connected to the Pacific s-LLVP at its southeastern tip. The shear-wave velocity reduction is slightly smaller than in model S40RTS, especially in the lowermost mantle (Supplementary Figure S4i,j).

The buoyancy number of SOC of 0.66 corresponds to a lower mantle excess density of $+4.5\%$ for basalt relative to the average mantle. This is slightly larger than estimates from mineral physics^44–46^. For a compressible mantle, this translates to a value of $+2.1\%$, similar to recent calculations of the excess density of SOC in the lower mantle^44,45^.

Nine additional simulations (Table 1) explore the effects of the assumed buoyancy of SOC in the lower mantle (B = 0.44 and B = 0.22)^44–46^, the mantle viscosity structure (visc2 and visc3)^47^, the CMB temperature (CMB2800 and CMB2600)^48,49^, the effects of compressibility (COMP), the presence of dense primordial material at the CMB (PRIM), and the choice of plate motion history (MER)^39^. With the exception of simulation visc2, all simulations have a total weighted layer correlation greater than the critical value of 0.3 up to degree 8 ($p=0.01$ from Becker and Boschi^50^) for depths greater than 1100 km (Supplementary Figure S5). Simulation MER has the lowest correlation to S40RTS through much of the lower mantle, which likely reflects differences in the plate motion reconstruction prior to 300 Ma.

## Evolution, composition, density and age differences between s-LLVPs

**Fig. 2:**
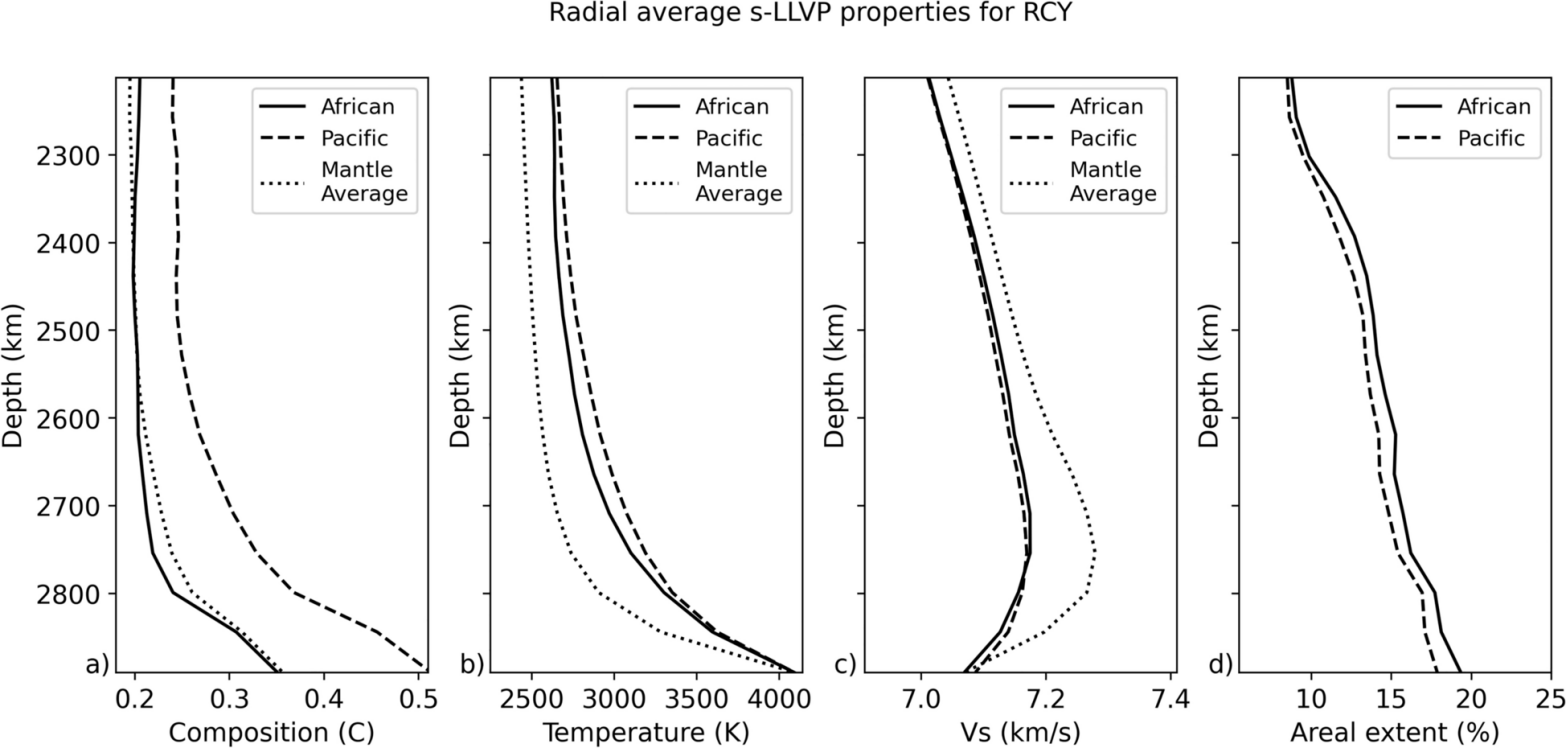
Radial average of present day s-LLVP properties in simulation RCY, showing (**a**) bulk composition, (**b**) temperature, (**c**) predicted absolute shear-wave velocity (unfiltered) in the African s-LLVP (solid lines) and Pacific s-LLVP (dashed lines) and (**d**) the areal extent of each s-LLVP in each radial layer. A theoretical adiabat (Supplementary Figure S6) has been added to the temperature field to account for model incompressibility.

To interrogate the African and Pacific s-LLVPs individually, we first isolate the low velocity regions using a K-means clustering technique applied to the filtered $\delta V_{s}$ field (Methods 6.5). We then separate the African and Pacific regions based on a longitude cut-off. The differences in the radially averaged bulk composition, temperature, and $V_{s}$ of the Pacific and African s-LLVPs in the lowermost 680 km of the mantle depend on the property considered. If measured by the depth-integrated areal extent of the s-LLVPs over each radial layer (Fig. 2d), the African s-LLVP is only 5% more voluminous than the Pacific s-LLVP (Fig. 2d). Their average temperature and shear-wave velocity (Fig. 2b,c) differ by up to only 3.9 and 0.18% respectively.

**Fig. 3:**
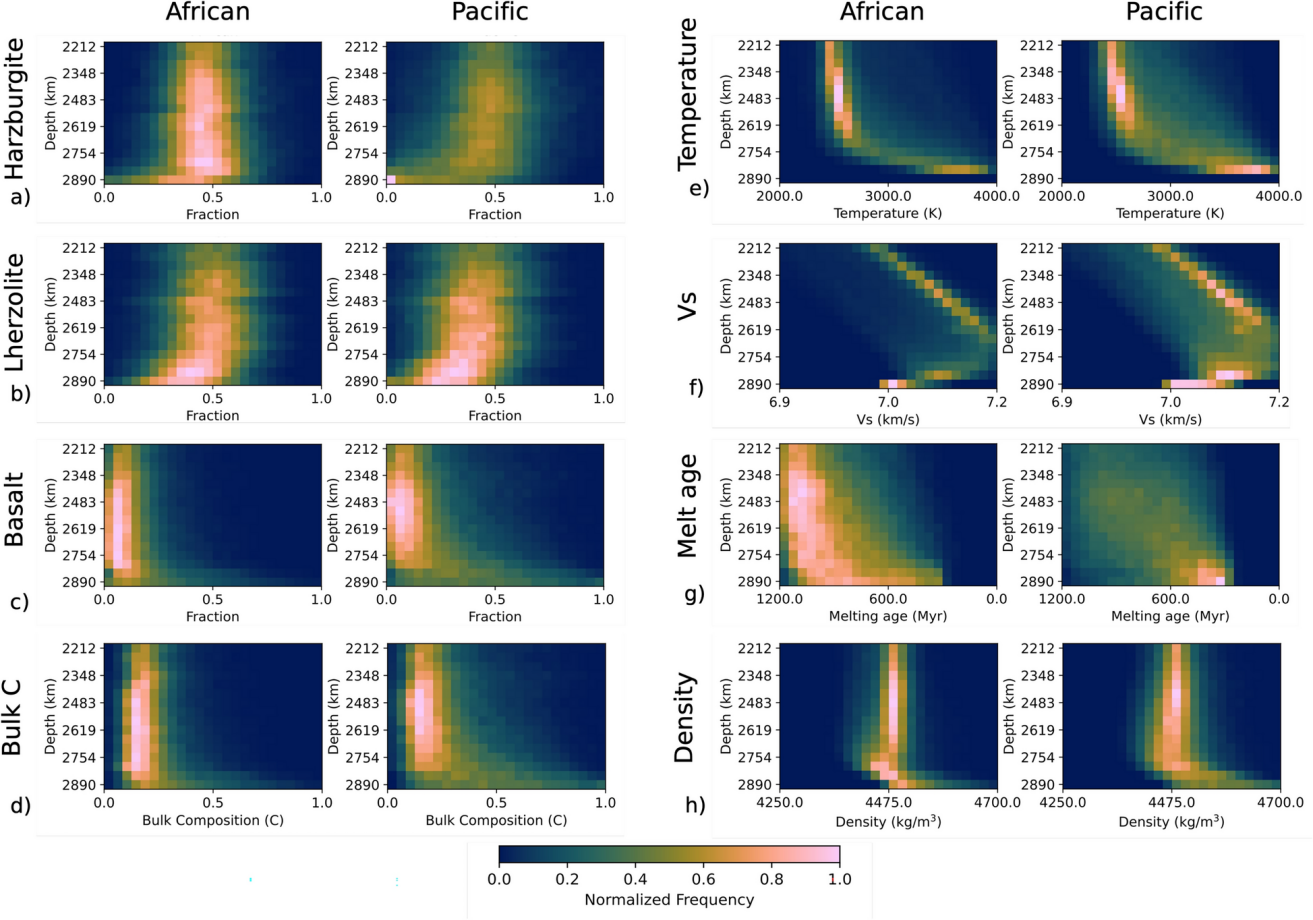
Histograms for bulk composition, temperature, shear-wave velocity ($V_{s}$, unfiltered), melting age and density of the African (left plot in each panel) and Pacific (right plot in each panel) s-LLVPs. The proportions of (**a**) harzburgite, (**b**) lherzolite, and (**c**) basalt together define the (**d**) bulk composition. We also plot the (**e**) temperature, (**f**) predicted absolute shear-wave velocity, (**g**) melting age and (**h**) density distributions. Due to the isothermal boundary condition of the simulation at the CMB, the temperature is not plotted at this depth.

However, the difference in composition is large. Above 2700 km depth, the bulk composition of the African s-LLVP is similar to the mantle average value of $C = 0.2$ (Fig. 2a), increasing to $C = 0.35$ at the CMB. In contrast, the Pacific s-LLVP contains a broader range of compositions (Fig. 3a–d), and it is enriched in basalt compared to the average mantle by up to 53%. Basalt enrichment of the Pacific s-LLVP is accompanied by reduced harzburgite and lherzolite fractions, with the harzburgite fraction being particularly small in the lowermost mantle (Fig. 3a). The compositional disparity between the Pacific and African s-LLVPs occurs in all simulations, although the degree of enrichment in SOC varies. For instance, in simulations with different radial viscosity profiles in the lower mantle (Supplementary Figure S7), the Pacific s-LLVP is enriched by up to 58% and 25% compared to the African s-LLVP for simulations visc2 and visc3 respectively. At lower CMB temperatures, the Pacific s-LLVP is also enriched relative to the African s-LLVP (Supplementary Figure S8), and the same difference is observed when using a compressible equation of state (Supplementary Figures S9, S3j–l). Even when using a plate reconstruction model that differs significantly before $\sim$ 300 Ma (Merdith et al.^39^, case ’MER’, Table 1), we find that the Pacific s-LLVP is strongly enriched in SOC compared to the African s-LLVP (Supplementary Figure S9e). This implies that SOC enrichment of the Pacific s-LLVP is a robust result in our simulations and indicates that the circum-Pacific downwellings during the past 300 Myr strongly influence the distribution of SOC in the lower mantle^15,30^.

The melting age, defined as the average time since particles in a cell underwent melting, offers an insight into the reworking history of s-LLVPs (Fig. 3g). For the African s-LLVP, the melting age distribution is mostly skewed towards older ages (high melting age), with greater proportions of younger (low melting age) material in the lowermost $\sim$ 150 km of the mantle. This implies that much of the constituent material of the African s-LLVP has been sequestered in the lower mantle for $\sim$ 1 Gyr. Conversely, the melting age distribution for the Pacific s-LLVP is a Gaussian centered on an age of 750 Myr down to $\sim$ 2600 km depth. At greater depths the melting ages are skewed to lower values (Fig. 3g). This suggests that on average the constituent material of the African s-LLVP is older than in the Pacific s-LLVP, and that there has been a large and recent influx of young SOC into the base of the Pacific s-LLVP. This may indicate that the African s-LLVP was formed prior to the Pacific s-LLVP or that in recent times young SOC accumulates preferentially in the Pacific s-LLVP.

**Fig. 4:**
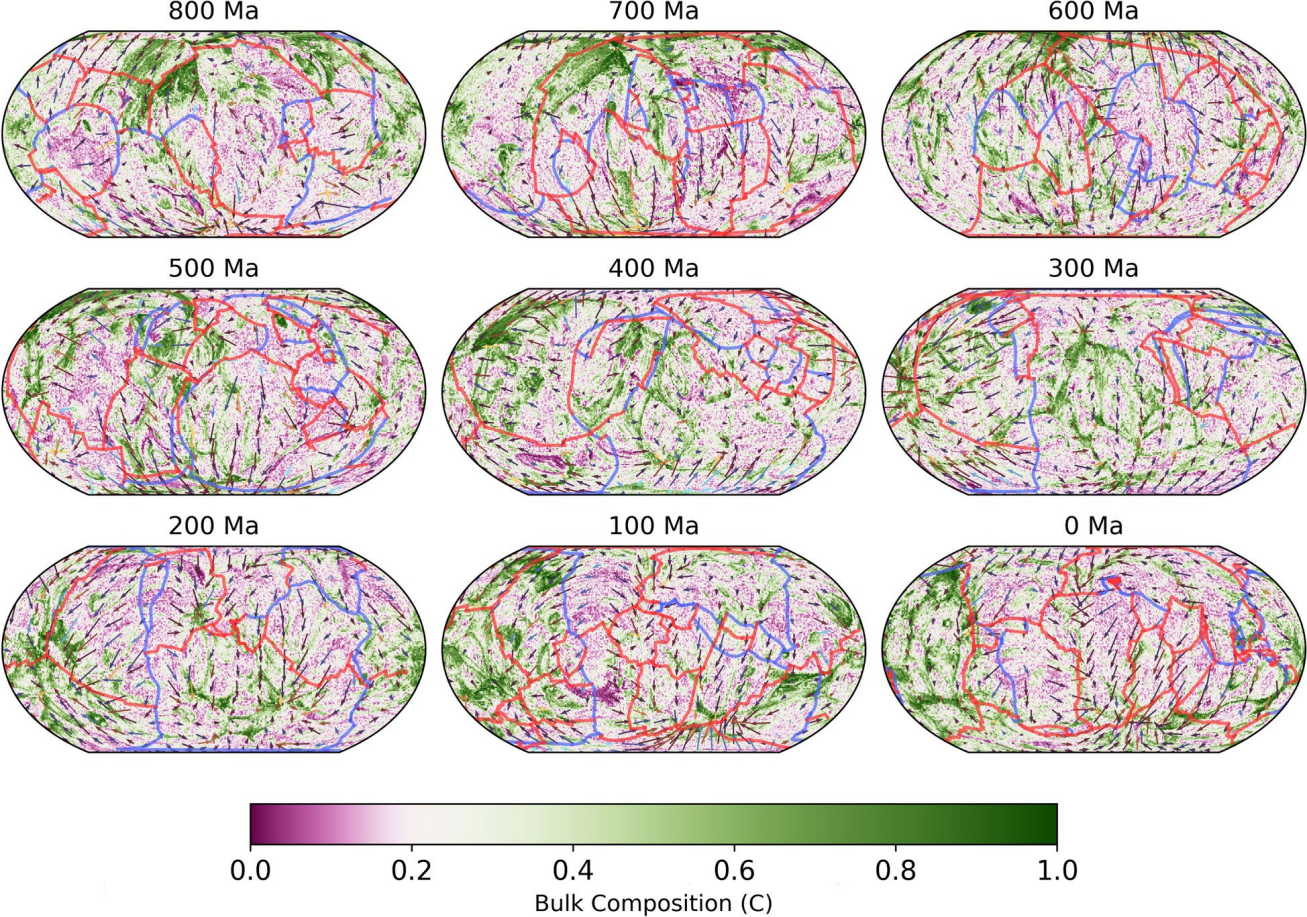
Time evolution of mantle composition, illustrated by depth slices at 2844 km depth at 100 Myr intervals from 800 Ma to present day for simulation RCY, coloured by bulk composition. Red lines indicate ridge and transform boundaries and blue lines indicate subduction zones, as defined by the plate reconstruction used for the surface boundary condition^38^. Arrows indicate the direction and magnitude of horizontal flow at 2844 km depth, coloured by the radial velocity with cool colours indicating downward flow (toward CMB) and hot colours indicating upward flow (toward surface).

**Fig. 5:**
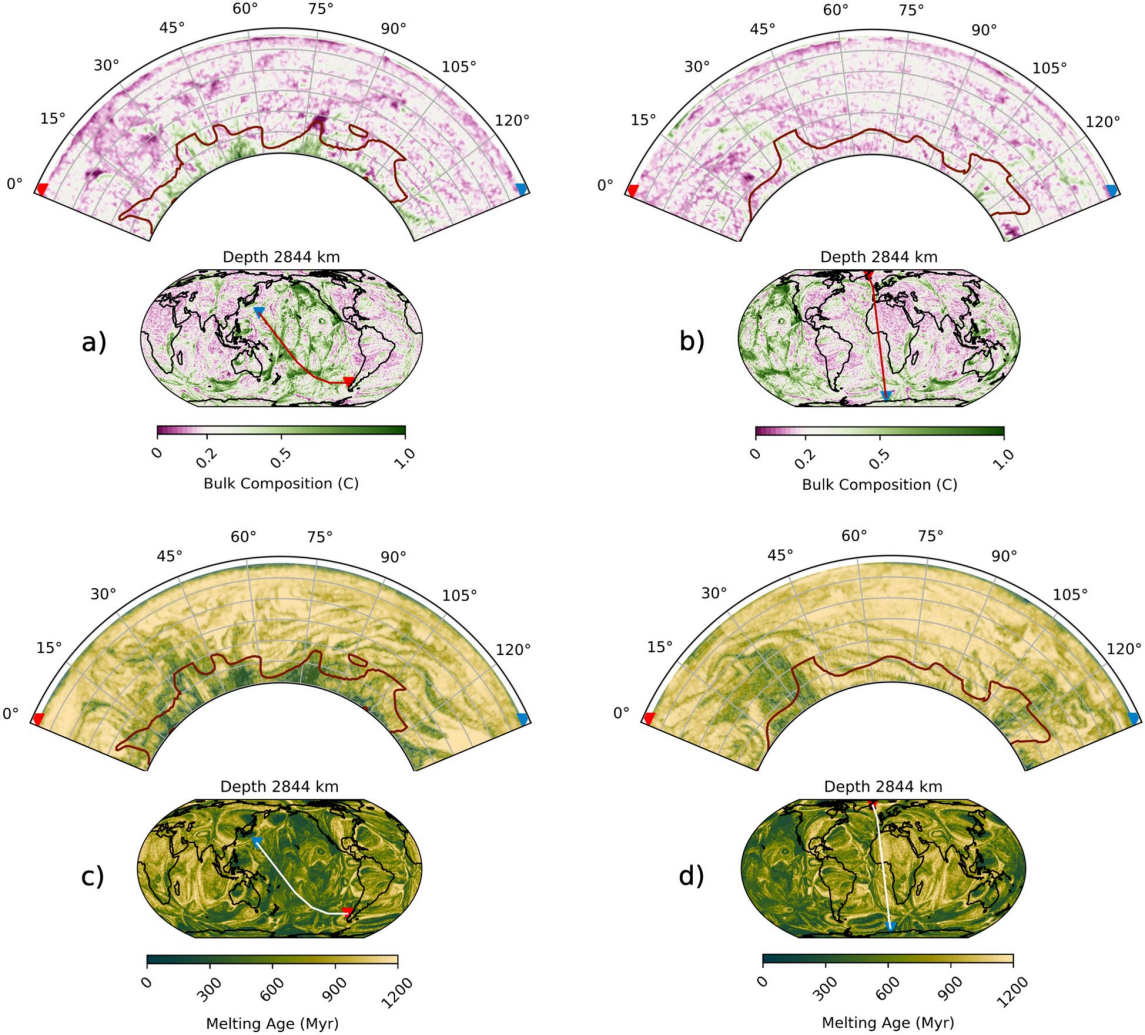
Cross sections through the present-day mantle as simulated by simulation RCY, coloured by (**a**,**b**) bulk composition and (**c**,**d**) melting age (i.e., average time since last melting). The inset map shows the location of the cross sections with red and blue triangles indicating the extent of the cross section. Cross section locations have been chosen to pass through (**a**,**c**) the Pacific s-LLVP and (**b**,**d**) the African s-LLVP. Outlines of the s-LLVPs are shown in red up to 1000 km above the CMB.

Simulation RCY predicts how the subducted oceanic crust accumulates (Fig. 4) and how it has been redistributed by mantle circulation over the past 1 Gyr. A large volume of SOC converges in the northern hemisphere from the beginning of the simulation until 800 Ma. Between 800 and 600 Ma, this volume begins to split while new SOC enters the lower mantle from subduction primarily at mid to low latitudes. From 600 to 400 Ma, the subduction zones migrate into a circum-planetary girdle and the accompanying downwellings push SOC away from these regions. At 300 Ma, strong lateral flow in the lowermost mantle brings SOC under the present-day Pacific region, while weak flow beneath present-day Africa allows SOC accumulations to move slowly south (Fig. 4, Supplementary Video SV1). From 200 Ma to present day, SOC continues to be added beneath the Pacific region, replacing the material that was lost through entrainment by persistent plumes, as implied by the melting age distribution (Fig. 3g). A steady rate of replenishment of young SOC^23,24^ therefore enables the Pacific s-LLVP to be maintained. Accumulations of SOC beneath present-day Africa continue to migrate south and are almost completely removed from the lowermost mantle by a strong plume that develops in the Southern Indian ocean. The entrained SOC is replaced by relatively old, depleted and well-mixed material compared to that which replenishes the Pacific s-LLVP (Figs. 4, 5).

In simulation RCY, the lower mantle excess density of basalt relative to the average composition ($+4.5\%$) is greater than current mineral physics estimates^44–46^. However, we find the same trend between the African and Pacific s-LLVP for simulations with buoyancy numbers that correspond to excess densities of $+3.0\%$ ($B=0.44$, Supplementary Figure S10e) and $+1.5\%$ ($B=0.22$, Supplementary Figure S10a), albeit with reduced basalt enrichment in the lowermost mantle. Due to a relatively high concentration of basaltic material, the Pacific s-LLVP is denser than the African s-LLVP (Fig. 3h), as SOC is expected to be denser relative to the ambient composition of the lowermost mantle. This intrinsic density may be a controlling factor on LLVP height, with the African LLVP being more buoyant and therefore more unstable^53^. Our results imply that the compositional, intrinsic density and height differences between the two LLVPs are a natural consequence of time-varying mantle flow and different time-integrated replenishment rates.

## Discussion

Our 3-D simulations of the production and circulation of oceanic crust using models of plate-motion history from the past billion years predict that SOC accumulates into two antipodal piles in the lower mantle beneath Africa and the Pacific akin to the two large low velocity provinces (LLVPs) imaged seismically (Fig. 4) [see also^10,15,30^]. Combined, the areal extent of the simulated s-LLVPs in RCY increases from 17% at 2212 km depth to 37% at the CMB (Fig.  2), making them laterally more extensive than seismically imaged LLVPs. This is likely due to the imperfect representation of historic subduction zones in the plate reconstruction model, inaccurate lower mantle viscosities, and the omission of the effects of composition-dependent viscosity from our models. The latter would affect the stability of basal mantle structures^54^, potentially making it easier for downwellings to sculpt the s-LLVPs and thus decreasing their footprint at the CMB. In our simulations, viscosity is only weakly temperature dependent and subducted slabs are therefore not very strong relative to the surrounding mantle. Reference^55^ showed that strong slabs may intermittently fold backwards onto themselves beneath the subducting plate, aiding segregation of oceanic crust to the CMB. Given that we observe high concentrations (Figs. 4, 5a) of young (Fig. 3g) SOC beneath the Pacific, where oceanic plates have been subducting for the last 300 Myr, it is possible that our simulations also feature this backwards folding effect, with circum-Pacific subduction preferentially feeding SOC to the Pacific s-LLVP. Stronger slabs could further enhance this effect, however there are other complexities such as grain-size and composition-dependent rheology to consider as well. It is therefore difficult to be certain about whether we accurately capture the slab morphologies. In this context, it is worth noting the match of our predicted seismic structures with S40RTS (Fig. 1i), which indicates that the subducted slabs are broadly located in the right geographic regions.

**Fig. 6:**
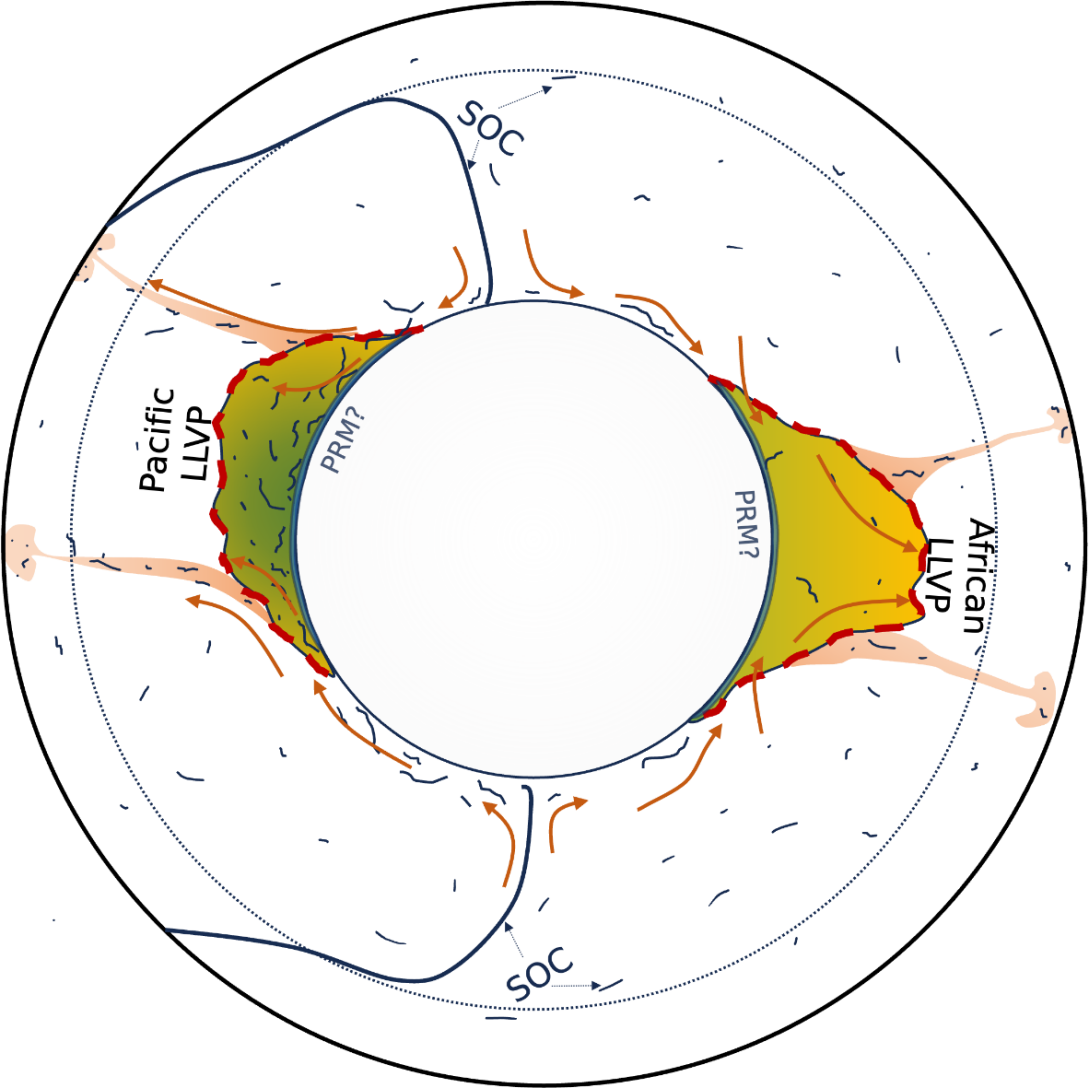
Schematic diagram for the proposed mechanisms that sustain the Pacific and African LLVPs over the past $\sim 300$ Myr. Dark blue strands represent SOC with orange arrows indicating the flow of material in and out of the LLVPs. The Pacific LLVP is fed at its base by young (green) SOC, while the African LLVP consists of well-mixed, older (yellow) material. Our simulations do not rule out the possibility of a thin layer of dense primordial material (PRM) at the base of the LLVPs.

Our simulations indicate that persistent plumes in the Pacific induced a mantle flow pattern at the CMB that pulled in newly subducted slabs (Supplementary Video SV1, Fig. 4, 6). This resulted in enrichment of the Pacific s-LLVP in young SOC (Fig. 5). In contrast, in the last $\sim$ 300 Myr the African s-LLVP is replenished with older, better mixed material with a bulk composition closer to the average mantle compared to the Pacific s-LLVP (Figs. 4, 5). In the plate reconstructions used, the conditions are favorable for persistent plumes in the Pacific during the last $\sim$300 Myr because subduction continues to occur around the margin of the Panthalassic Ocean after the assembly of Pangea up to present day as it morphs into the modern Pacific Ocean^38^.

The enrichment in basalt of the Pacific s-LLVP appears inconsistent with recent geochemical inferences that enriched mantle type intra-plate hotspots are more commonly associated with the African LLVP^56^. However, the difference in mean bulk composition of plumes associated with the two s-LLVPs is surprisingly low in RCY (Supplementary Figure S11), given the compositional difference between the two s-LLVPs (Fig. 2a). This may be because plumes form and preferentially entrain material from the edges of the s-LLVPs^57^. The mean plume composition of the two domains also converges towards the surface, possibly due to increased mixing at shallower depths. It should be noted that our simulations do not include recycling of continental crust or marine sediments, so we do not expect to explain all of the compositional complexities observed in mantle plumes.

Some studies suggest that the African LLVP reaches up to 550km higher above the CMB than its counterpart^7,8^ although the exact height difference is subject to the tomographic model and velocity contour used to define the LLVPs. Contrasting internal buoyancy between the LLVPs caused by different compositions^53^ and distinct formation histories has been suggested as a reason for the height difference. Geochemical evidence from an analysis of plume lavas associated with the two regions^56^ appears to support a compositional difference between the African and Pacific LLVPs. However, others suggest that apparent geochemical differences are due to sampling bias and argue that there is no significant difference between the isotopic composition of plumes originating from the African and Pacific LLVPs^58^, with height differences between the LLVPs being attributed to limited seismic resolution^59,60^ and uncertain image interpretations. Our simulations suggest that compositional differences between the LLVPs are expected to naturally arise due to the historic pattern of subduction of oceanic crust. Consequently the internal density of structure may differ between the two LLVPs (Fig. 3h), contributing to their height difference.

Despite the strong compositional contrast between the African and Pacific s-LLVPs (Figs. 2a, 3a–c), their predicted radially averaged temperature and $V_{s}$ differ by only 3.9% and 0.18% respectively (Figs. 2b,c, 3e,f). This is because of the dominant effect of temperature relative to composition on the $V_{s}$ of lower mantle minerals. While this arises from the mineral physics dataset used in our study (Methods 6.3, Supplementary Figure S12), we believe the conclusion that the temperature effect dominates that of the composition holds up in general^61^.

Recent work suggests that in order to fit multiple robust geophysical constraints, such as dynamic topography, the geoid and normal-mode frequency variations, LLVPs may contain denser than average material at their base^5^, with chondrite-enriched basalt (CEB) proposed as a possible composition for this material^12^. Since our simulation is limited to the past billion years for which plate reconstruction models are available, it is not feasible to incorporate the recycling of CEB during the Hadean in our simulations. Instead, we can follow a similar approach as employed in many other modeling studies to simulate a dense primordial layer and use this to investigate an end-member scenario for the early recycling of oceanic crust. We initialise the model with a 150 km thick layer of intrinsically dense material with a unique ($C = 2.0$) composition at the CMB at 1.2 Ga (simulation PRM, Table 3). We implicitly assume that this layer represents CEB material that was produced and segregated to the CMB prior to 1.2 Ga. We find that the Pacific s-LLVP remains more enriched in SOC than the African s-LLVP (Supplementary Figure S13c), while it is additionally enriched in the $C = 2.0$ component (Supplementary Figure S13d). At the base of the mantle, this amplifies $\delta V_{s}$, making it similar to that observed in S40RTS (Supplementary Figure S4i), compared to simulation RCY that underestimates the shear-wave velocity reduction (Supplementary Figure S4a-f). However, at shallower depths the velocity reduction in PRM is larger than observed in S40RTS (Supplementary Figure S4g,h). Given the more Earth-like shear-wave velocity reductions that can be achieved at the base of the s-LLVPs when they are enriched in CEB, we cannot rule out that primordial, chemically distinct material is present at the base of LLVPs, possibly buried and trapped at the base of the mantle by SOC^62,63^. The results of simulation PRM suggest that SOC that has segregated at the CMB prior to 1.2 Ga can remain there until present day. We note, however, that the initial distribution of primordial material (simulated by an uniform layer above the CMB in PRM) is likely unrealistic, and our results thus only apply to the specific modeled scenario. As the other simulations presented in this study do not account for SOC that has segregated at the CMB prior to model initialisation, the present day distributions that they predict must be considered to only reflect SOC material produced after 1.2 Ga. We expect that this limitation is less important for the Pacific domain, where we have found that the s-LLVP is strongly enriched in young SOC (Fig.  3g), and note that the African domain had previously be enriched in SOC, which has since been entrained away (Fig. 4).

In the broadest sense, the predicted distributions of SOC within the s-LLVPs depend on its segregation into the lowermost mantle and subsequent entrainment. The resolution of the geodynamic model is therefore important, as the oceanic crust is thin (6–7 km) relative to the thickness of the mantle. While our model grid is not fine enough to resolve Earth-like oceanic crust, the particles used for tracing composition offer a sub-grid resolution, estimated to give a mean oceanic crust thickness of 10 km (“Particles”). This oceanic crust is expected to largely follow the trajectory of the subducting slab as it descends into the mantle due to viscous coupling^35^, and we thus expect the large-scale distribution of SOC to be unaffected by the limited resolution of our model grid. Nevertheless, we cannot avoid the issue that the coarse resolution of our simulations may not fully capture the dynamics of slabs in the mantle and may therefore over-estimate the rate of entrainment^35^.

The assumption of an incompressible mantle may contribute to the relatively small reduction in shear-wave velocity of the s-LLVPs compared to tomographic observations. To account for compressibility of the mantle, we add a theoretical adiabat to the simulations, scaling the temperature field at each depth by the same value. While this process leads to 1D mantle temperature profile with a more Earth-like thermal gradient, the temperature range at each depth is unchanged. This may lead to reduced lateral variation in shear-wave velocity compared to a compressible mantle circulation simulation because a compressible mantle could retain cooler slabs relative to those in the temperature adjusted incompressible model. However, our tests using a compressible mantle circulation simulation (COMP), indicate that the velocity reductions in the s-LLVPs are not significantly different from those in the reference simulation RCY (Supplementary Figure S4d,h,l). Like RCY, the compressible simulation produces a Pacific s-LLVP that is enriched in SOC compared to the African s-LLVP (Supplementary Figure S9a). A further source of error in estimating seismic velocities stems from the choice of the thermodynamic database. Although we have used a recent version of an established database^64^, this does not include the iron spin transition in the lower mantle^65–67^ and it differs from an earlier, widely-used version^68^. The significant changes in thermodynamic databases reflect the fact that the determination of seismic velocities at lower mantle pressures and temperature remains difficult due to experimental challenges under extreme conditions.

## Conclusions

Our models of mantle circulation over the past billion years demonstrate that LLVPs can naturally develop as a consequence of recycling oceanic crust. The African and Pacific LLVPs have similar elevated temperatures and hence similar reduced shear-wave speeds since temperature affects $V_{s}$ more than composition. However, the Pacific and African LLVP evolve differently and have different compositions as a consequence of the plate motion history from the past 1 Gyr. Melting ages indicate that the material in the African LLVP is older and better mixed than in the Pacific LLVP which has been replenished by subducted oceanic crust during the past 300 Myr. Compared to the Pacific LLVP, the African LLVP is less enriched in basaltic material and therefore less dense and more buoyant, supporting the idea that the African LLVP can rise higher into the mantle than the Pacific LLVP. Further studies should investigate the additional role of primordial material at the base of LLVPs on their dynamical evolution.

## Methods

### Dynamic simulations

The simulations presented in this study have been run using the three-dimensional mantle convection code, TERRA^36,37,69,70^. Under the Boussinesq approximation and assuming an incompressible mantle^71^, the equations for conservation of mass, momentum and energy are:

1 $$\begin{aligned} & \nabla \cdot \varvec{u}=0 \end{aligned}$$

2 $$\begin{aligned} & \nabla \cdot \left( \eta \left\{ \nabla \varvec{u}+(\nabla \varvec{u})^T\right\} \right) -\nabla p = \varvec{g} \hspace{0.1em} \alpha \hspace{0.1em} \rho _{0} \hspace{0.2em} (\delta T - C \hspace{0.1em} B \hspace{0.1em} \Delta T) \end{aligned}$$

3 $$\begin{aligned} & \rho _{0} \hspace{0.2em} C_{p} \left( \frac{\partial T}{\partial t}+\textbf{u} \cdot \nabla T \right) - k \hspace{0.2em}\nabla ^2 T - H=0 \end{aligned}$$

respectively, where $\varvec{u}$ is fluid velocity, $\eta$ is dynamic viscosity, *T* is temperature, *p* is dynamic pressure, $\varvec{g}$ is the acceleration due to gravity, $\alpha$ is the coefficient of thermal expansion, $\rho _{0}$ is the reference density, *C* is the bulk composition (ranging between 0 and 1), *B* is the buoyancy number, $\Delta T$ is the temperature contrast across the mantle, *t* is time, $C_{p}$ is the specific heat capacity constant pressure, *k* is thermal conductivity, *H* is the radiogenic heat production per unit volume and $\delta t = (T - T_{ref})$ where $T_{ref}$ is our reference temperature profile. The buoyancy number is defined as:

4 $$\begin{aligned} B=\frac{\Delta \rho _{b}}{\alpha \rho _{0} \Delta T} \end{aligned}$$

where $\Delta \rho _{b}$ is the intrinsic density difference between basalt ($C = 1$) and lherzolite ($C = 0.2$, average mantle) in the lower mantle. The advection of bulk composition is described as:

5 $$\begin{aligned} \frac{\partial C}{\partial t} = -\nabla \cdot (C \textbf{u}) + S \end{aligned}$$

as in van Heck et al.^40^, where *S* represents melting described in detail below in “Particles”. The model parameters and parameter values are listed in Tables 1 and 2.

The model domain is discretised into 65 concentric layers, each composed of a regular icosahedron that is projected onto a sphere, with a radial spacing of $\sim$45 km. At each radial layer, the icosahedron is sub-divided, leading to an average lateral resolution at the surface and CMB of $\sim$60 km and $\sim$33 km respectively^36^. While such a resolution is sufficient to resolve long-wavelength mantle features, it is too large to resolve the $\sim$ 6–7 km thick oceanic crust. The oceanic crust in our models is defined by bulk composition, which is tracked on particles, thus providing a sub-grid resolution. To estimate the thickness of the oceanic crust we look at the fraction of particles (“Particles”) in the oceanic domain that have a bulk composition of $C = 1.0$ in the oceanic basins. As oceanic crust is by definition basaltic, at least more than 50% of particles at a given depth must be basaltic ($C = 1.0$). Using this definition, we find the oceanic crust to have an average thickness of 10 km in our simulations (Supplementary Figure S14). This includes any unusually thick areas of crust that could be likened to plateaus and seamounts. This oceanic crust is expected to largely follow the trajectory of the subducting slab as it descends into the mantle due to viscous coupling^35^.

**Table 2:** Common parameters to all simulations and their values. Reference viscosity is equal to the viscosity of the upper mantle.

Symbol	Parameter	Value	Unit
$T_{s}$	Surface temperature	300	*K*
$\eta _{0}$	Reference viscosity	$4 \times 10^{21}$	$Pa \hspace{0.2em} s$
$\rho _{0}$	Reference density	4500	$kg \hspace{0.2em} m^{-3}$
*k*	Thermal conductivity	4	$W \hspace{0.2em} m^{-1} K^{-1}$
$\alpha$	Thermal expansivity	$2.5 \times 10^{-5}$	$K^{-1}$
$C_{p}$	Specific heat capacity	1100	$J \hspace{0.2em} kg^{-1} K^{-1}$

From an initial temperature distribution, the simulation is allowed to evolve for 2 Gyr with a free-slip surface boundary condition in a pre-conditioning phase. This ensures that any signal from the initial thermal structure is removed. We then run the conditioning phase whereby the first stage of the plate motion reconstruction^38^ acts as the surface velocity boundary condition. This is applied for 200 Myr in order to introduce temperature, velocity and compositional structures into the mantle that reflect the overlying plate assemblage. Finally, each simulation is run forwards in time from 1000 Ma to the present day with surface velocities updated every 1 Myr.

To avoid numerical instabilities and artefacts, the reference viscosity used in our simulations (Table 2), is higher than what is expected for Earth’s upper mantle by approximately a factor of 4. Consequently, the RMS surface velocities during the pre-conditioning phase of the simulations ($\sim$ 2.5 cm/year), prior to plate velocities being imposed, are about 1/2 of what is estimated for the present day RMS surface velocity for plates on Earth^38^. Therefore, we apply a scaling factor of 1/2 to the reconstructed plate velocities so that they better match the flow velocities in our simulations, i.e. the reconstructed plate velocities are reduced by 50%. To compensate for the reduced surface velocities and to maintain the correct volume flux of material through ridges and trenches, we must then apply a scaling factor of 2 to the model time *t*^72^. This gives rise to an important distinction between ‘model time’ and ‘Earth time’. Throughout this work, we quote time as Earth time when it is directly related to geological time, whereas the model time is larger by a factor of 2 (the time scaling factor). While the plate reconstructions define the surface velocities, they do not control the polarity of subduction in our simulations. Without an imposed polarity of subduction, simulations with a free-slip surface boundary condition exhibit two-sided subduction, with slabs sinking vertically into the mantle^73^. However, due to the retreat of subduction zones in the assimilated plate velocities, we do observe single-sided subduction behaviour, with slabs primarily entering the mantle with an Earth-like dip and polarity. Imposing the correct subduction polarity (as in Bower et al.^14^) would likely improve the shallow structure in subduction settings.

Viscosity in the simulations depends on depth and temperature according to:

6 $$\begin{aligned} \eta =\eta _{z} \exp ((z{'}V_{a}) - (E_{a}T{'})) \end{aligned}$$

where $\eta$ is the viscosity, $\eta _{z}$ is the reference viscosity ($\eta _{0}$) multiplied by the radial viscosity factor (Supplementary Figure S1) at depth *z*, $z{'}$ is the non-dimensional depth, $V_{a}$=1.0 and $E_{a}$=2.0 are non-dimensional constants that control the sensitivity of viscosity to depth and temperature, and $T{'}$ is the non-dimensional temperature. Depth is non-dimensionalised by $z{'} = z / h$, where *h* is the thickness of the mantle. Temperature is non-dimensionalised by $T{'} = (T - T_s) / (T_c - Ts)$, where *T* is the mantle temperature at a given point, $T_s$ is the temperature of the surface boundary, and $T_c$ is the temperature of the lower boundary at the CMB.

All profiles for $\eta _{z}$ feature a strong lithosphere with a thickness of 135 km, a weak upper mantle with viscosity equal to the reference value, and a 30$\times$ viscosity jump across the 660-km discontinuity^74^. In the lower mantle, we use three profiles (Supplementary Figure S1) to explore different causes for an increase of the viscosity with depth, assuming it to be either due to increasing bridgmanite concentrations or increasing strength of ferropericlase with depth. In the lowermost mantle the radial viscosity profile also features a reduction to approximate the decrease in viscosity associated with the lower mantle bridgmanite to post-perovskite phase transition^31^ and the CMB thermal boundary layer. The impact of the latter is also accommodated by the temperature dependence of the viscosity.

### Particles

#### Bulk composition parameterization

We use tracer particles to track bulk composition and abundance of heat producing elements. Simplified bulk composition is stored as a value (*C*) that varies between $C = 0.0$, representing completely depleted material (harzburgite), and $C = 1.0$, representing completely enriched material (basalt), while we consider the bulk silicate Earth composition to be lherzolite. Together, these three compositions represent the characteristic lithologies of the mantle. They are each assigned a bulk composition composed of six major oxides (Table 3), with proportions chosen to fit results from Baker and Beckett^75^ for harzburgite, Walter^76^ for lherzolite and White and Klein^77^ for basalt. To determine the *C*-value of lherzolite, we find the best fit vector between the major element mass proportions of harzburgite, lherzolite and basalt, finding this to be $C = 0.2$. Different choices for the bulk composition of basalt, harzburgite and lherzolite result in slightly different values of *C* (ca. 0.18–0.21 mass fraction basalt).

At the start of the pre-conditioning phase, each cell is initialised with 10 particles, with 1 $\times$ $C = 1.0$ particle, 5 $\times$ $C = 0.2$ particles and 4 $\times$ $C = 0.0$ particles, giving a mean mantle composition of $C = 0.2$.

**Table 3:** Assumed molar composition for our three standard characteristic lithologies (harzburgite, lherzolite, basalt) as well as the ’primitive’ composition (CEB).

	Harzburgite	Lherzolite	Basalt	CEB
SiO2	36.184	38.819	52.298	48.47
MgO	56.559	49.894	15.812	20.00
FeO	5.954	6.145	7.121	11.28
CaO	0.889	2.874	13.027	10.59
Al2O3	0.492	1.963	9.489	11.28
Na2O	0.001	0.367	2.244	1.50

#### Solid phase transitions in the geodynamic simulations

In our geodynamic simulations, we use a simplified parameterization of the phase transitions in the olivine system, which occur at 410 and 660 km depth (Table 4). This parameterization allows us to reproduce some of the behaviour associated with these discontinuities^41,78^. The depth and bulk composition of particles affects the density field, which is quantified by the buoyancy number (Eq. 4). We vary the buoyancy number in our simulations (Table 1) to investigate the effect of different intrinsic densities of SOC^24^, estimates of which vary between 0.5 and $5\%$ more dense than average mantle^44,46,79^.

#### Melting in the geodynamic simulations

A linear solidus, dependent on depth (*z*) and bulk composition, controls melting in the simulations^24,40,41^:

7 $$\begin{aligned} T_{\text{ solidus }}(z, C)=T_{\text {meltsurf}}+z T_{\text {meltslope}}+(1-C) T_{\text {meltcomp}} \end{aligned}$$

where $T_\text {meltsurf}$ = 1200 K is the melting temperature of basalt ($C = 1$) at the surface, $T_\text {meltslope}$ = 2.5 K $\hbox {km}^{-1}$ is the gradient of the solidus and $T_\text {meltcomp}$ = 500 K is the temperature difference between the solidi of basalt ($C = 1$) and harzburgite ($C = 0$). At each time step, we check whether particles in the uppermost 135 km have crossed their solidus. If this is the case, the melting particle has its bulk composition reduced so that it plots on the solidus for the particle’s temperature and pressure until it cannot be further depleted ($C=0$). Particles in the shallowest cell vertically above the melt producing particle are enriched with the produced melt first, assuming instantaneous melt migration. If all particles in the shallowest cell are completely enriched, subsequently particles in the cell below are checked to see whether they can be enriched by the melt. Full details of this melting process in the dynamic simulations can be found in refs.^24,40,42^.

**Table 4:** Olivine phase change parameters for an assumed composition with 67% $\text {(Mg,Fe)}_{2} \text {SiO}_{4}$.

Depth (km)	${\Delta \rho }$ kg $\hbox {m}^{-3}$	Clapeyron slope MPa $\hbox {K}^{-1}$
410	230	2.25
660	380	$-1.5$

### Seismic properties

#### Converting from simulation to seismic properties

We convert the pressure, temperature and composition of our simulations to seismic properties using look-up tables for each of the characteristic lithologies. Due to the incompressible equation of state used in our simulations, we add a theoretical adiabat to the simulated temperature field before performing this conversion. We use the thermodynamic data set of^64^, implemented in the Perple_X software^80^ to generate tables for density and effective isotropic seismic properties for each lithology in pressure - temperature space. Attenuation is accounted for using model Q7g^81,82^. The thermodynamic data set includes all major mantle phase transitions, but does not include the spin transition in ferropericlase, nor the second order phase transition in stishovite. Although these may reduce seismic velocities in the lower mantle, mineral physics studies indicate that the effect on the shear-wave velocity is small^67^.

As the bulk composition in our simulation is tracked using only a single parameter *C*, it is not possible to differentiate between mechanical and equillibrated mixtures of different compositions. We therefore make a pragmatic decision for how to convert *C* to lithology. An important requirement is that primitive mantle is modelled as pure lherzolite, not as a mechanical mixture of basalt and harzburgite. Therefore, we model particles with a bulk composition between $C = 0.0$ and $C = 0.2$ as a mechanical mixture^83^ of harzburgite and lherzolite, and particles with a bulk composition between $C = 0.2$ and $C = 1.0$ as a mechanical mixture of lherzolite and basalt. The relative proportions of each characteristic lithology are interpolated from the particles to the grid so that at each grid point we have information on the relative proportions of each of the three lithologies. Seismic properties at the grid point are then calculated by taking the harmonic mean of the properties for each lithology at the temperature and pressure of the grid point, weighted by the relative proportions of each lithology. Although our one-parameter compositional tracking and simplified geodynamic approach to phase transitions do not capture the full effects of chemical variation in the mantle, our post-processing approach is highly efficient and facilitates a detailed exploration of model parameters such as buoyancy number (excess density of SOC), independently from the thermodynamic model. This also allows us to investigate the choice of different assumed compositions independently of the geodynamic model.

#### The dominant effect of temperature on $V_{s}$

For the assumed molar compositions of the characteristic lithologies (Table 3), the predicted $\delta V_{s}$ between lherzolite and harzburgite is small (Supplementary Figures S12a,b,d, S15) at lower mantle pressures. This is because both lherzolite and harzburgite are silica-undersaturated and are dominated by bridgmanite and ferropericlase. The $V_{s}$ of basalt varies more strongly with temperature and pressure (Supplementary Figure S12c). Between 80 and 95 GPa, the $V_{s}$ of basalt is about 0.1 km/s higher than that of lherzolite (Supplementary Figure S12e) and harzburgite (Supplementary Figure S12f), but with increasing pressure the $V_{s}$ of basalt becomes lower than lherzolite and harzburgite across an increasingly wide temperature range. Nonetheless, the average difference in absolute $V_{s}$ between harzburgite and basalt is just 0.07 km/s (about 1%). However, within the temperature range relevant to the s-LLVPs (2200–4300 K, Fig. 3d), the mean of the differences between the maximum and minimum $V_{s}$ at each 0.1 GPa pressure point between 80 and 140 GPa is 0.42 km/s for harzburgite and 0.40 km/s for lherzolite and basalt, i.e. about 5% variation.

As such, velocity variations are primarily determined by temperature variations in the mantle since the 5% variations in $V_s$ due to temperature are greater than the 1% variations arising from compositional differences. The $\delta V_{s}$ of the two s-LLVPs is thus comparable because they are both similarly hot compared to the ambient mantle^13,84^ (Fig.2b). Compositional differences (Figs. 3a–c, 2a) are undetectable seismically (Fig. 2c), especially if the post-perovskite transformation is suppressed (Supplementary Figure S16). Note that the precise details of the post-perovskite transition remain debated.

### Filtering

In order to quantitatively compare the numerical simulations to seismic tomography, we follow the approach of^59^, thus accounting for the limited tomographic resolution. We choose to compare our simulations to seismic tomography model S40RTS as it includes good coverage in the lower mantle and the resolution matrix is readily available, which is essential for filtering the simulation results. The maps of simulated shear wave velocity variations in the mantle are first re-parameterized to spherical harmonic coefficients up to degree 40 and the same 21 radial splines as in model S40RTS. We then apply the S40RTS resolution matrix that describes how the tomographic resolution varies spatially due to the non-uniform seismic data coverage and applied model damping. The tomographic filter smooths the seismic velocity variations and suppresses amplitudes, but makes our predictions based on the simulation directly comparable with S40RTS, for example in Fig. 1 and Supplementary Figure S4, which show the filtered $\delta V_{s}$ field.

We compute the correlation between the geodynamic model and the seismic tomography model (Supplementary Figure S3) directly using their spherical harmonic expansions. Suppose that for degree *l* and order *m*, the coefficients of the geodynamic model are $\{a_{lm},b_{lm}\}$ and for the seismic tomography model they are $\{c_{lm},d_{lm}\}$. The depth-dependent degree correlation between the two models is then given by^50,85^:

8 $$\begin{aligned} r^l (z)=\frac{\sum _{m=0}^l\left( a_{d l m} c_{d l m}+b_{d l m} d_{l m}\right) }{\sqrt{\sum _{m=0}^l\left( a_{l m}^2+b_{l m}^2\right) } \sqrt{\sum _{m=0}^l\left( c_{l m}^2+d_{l m}^2\right) }}, \end{aligned}$$

where we have omitted the depth dependence of $a_{lm}$, $b_{lm}$, $c_{lm}$ and $d_{lm}$. We further calculate the total weighted layer correlation up to degree $l_{max}$ ($r_{l_{\text{ max } }}^{t o t}$) using:

9 $$\begin{aligned} r_{l_{\text {max }}}^{t o t}=\frac{\sum _{l=1}^{l_{\max }} \sum _{m=0}^l\left( a_{l m} c_{l m}+b_{l m} d_{l m}\right) }{\sqrt{\sum _{l=1}^{l_{\max }} \sum _{m=0}^l\left( a_{l m}^2+b_{l m}^2\right) } \sqrt{\sum _{l=1}^{l_{\max }} \sum _{m=0}^l\left( c_{l m}^2+d_{l m}^2\right) }}. \end{aligned}$$

### Identifying low velocity domains in our simulations

To identify low velocity domains in the filtered $V_{s}$ field predicted from our simulations, we use a K-means clustering algorithm^86^. We apply this to the filtered $\delta V_{s}$ field at depths between 2212 - 2890 km depth to split the field into three clusters. The points in the ‘low’ cluster comprise the simulated large-low-velocity-provinces (s-LLVPs). We separate this low cluster into the African and Pacific domains based on their longitude, where points in the Pacific domain have longitude $> 140^{\circ }$ and $< -50^{\circ }$. The geographic positions of these points in the model domain are then used to mask the model output fields to extract data only for points within the Pacific, African or both s-LLVPs (Supplementary Figure S2).

## Supplementary Information

Supplementary Information.

## Data Availability

Simulation outputs for this study can be accessed at 10.5281/zenodo.14187340.

## Code availabilty

The code TERRA used in this study is not freely available as the code predates open-source licensing. As such we do not have the rights to release all parts of the code, however code pieces which have been implemented for this study are available at 10.5281/zenodo.14187340 along with code for reproducing analysis carried out in this study. We also make use of the terratools package (10.5281/zenodo.11506398) for the analysis of simulation results. The Perple$\_X$ code to generate mineral physics lookup tables^80^ is available at www.perplex.ethz.ch/. The code used for tomographic filtering of model results^59^ can be requested viawww.earth.ox.ac.uk/$\sim$niv4152/downloads_filtering.html.

## References

[1] Garnero, E. J., McNamara, A. K. & Shim, S.-H. Continent-sized anomalous zones with low seismic velocity at the base of Earth’s mantle. *Nat. Geosci.* **9**(7), 481–489. 10.1038/ngeo2733 (2016).

[2] Li, X.-D. & Romanowicz, B. Global mantle shear velocity model developed using nonlinear asymptotic coupling theory. *J. Geophys. Res. Solid Earth* **101**(B10), 22245–22272. 10.1029/96JB01306 (1996).

[3] Ritsema, J., Heijst, H. J. V. & Woodhouse, J. H. Complex shear wave velocity structure imaged beneath Africa and Iceland. *Science* **286**(5446), 1925–1928. 10.1126/science.286.5446.1925 (1999). 10583949 10.1126/science.286.5446.1925

[4] Ishii, M. & Tromp, J. Normal-mode and free-air gravity constraints on lateral variations in velocity and density of Earth’s mantle. *Science* **285**(5431), 1231–1236. 10.1126/science.285.5431.1231 (1999). 10455043 10.1126/science.285.5431.1231

[5] Koelemeijer, P., Deuss, A. & Ritsema, J. Density structure of Earth’s lowermost mantle from Stoneley mode splitting observations. *Nat. Commun.* **8**(1), 15241. 10.1038/ncomms15241 (2017). 28504262 10.1038/ncomms15241 PMC5440685

[6] Cottaar, S. & Lekic, V. Morphology of seismically slow lower-mantle structures. *Geophys. J. Int.* **207**(2), 1122–1136. 10.1093/gji/ggw324 (2016).

[7] Wang, Y. & Wen, L. Geometry and P and S velocity structure of the African Anomaly. *J. Geophys. Res. Solid Earth.*[SPACE]10.1029/2006JB004483 (2007).

[8] He, Y. & Wen, L. Structural features and shear-velocity structure of the Pacific Anomaly. *J. Geophys. Res. Solid Earth.*[SPACE]10.1029/2008JB005814 (2009).

[9] Koppers, A. A. P. et al. Mantle plumes and their role in Earth processes. *Nat. Rev. Earth Environ.* **2**(6), 382–401. 10.1038/s43017-021-00168-6 (2021).

[10] Cao, X., Flament, N., Bodur, Ö. F. & Müller, R. D. The evolution of basal mantle structure in response to supercontinent aggregation and dispersal. *Sci. Rep.* **11**(1), 22967. 10.1038/s41598-021-02359-z (2021). 34824342 10.1038/s41598-021-02359-z PMC8617165

[11] Jackson, M. G., Becker, T. W. & Konter, J. G. Geochemistry and distribution of recycled domains in the mantle inferred from Nd and Pb isotopes in oceanic hot spots: Implications for storage in the large low shear wave velocity provinces. *Geochem. Geophys. Geosyst.* **19**(9), 3496–3519. 10.1029/2018GC007552 (2018).

[12] Richards, F. D., Hoggard, M. J., Ghelichkhan, S., Koelemeijer, P. & Lau, H. C. P. Geodynamic, geodetic, and seismic constraints favour deflated and dense-cored LLVPs. *Earth Planetary Sci. Lett.* **602**, 117964. 10.1016/j.epsl.2022.117964 (2023).

[13] Davies, D.R., Goes, S., & Lau, H.C.P. Thermally Dominated Deep Mantle LLSVPs: A Review. In: Khan, A., Deschamps, F. (eds.) The Earth’s Heterogeneous Mantle: A Geophysical, Geodynamical, and Geochemical Perspective, pp. 441–477. Springer, Cham (2015). 10.1007/978-3-319-15627-9_14

[14] Bower, D. J., Gurnis, M. & Seton, M. Lower mantle structure from paleogeographically constrained dynamic Earth models. *Geochem. Geophys. Geosystem.* **14**(1), 44–63. 10.1029/2012GC004267 (2013).

[15] Grabreck, A., Flament, N. & Bodur, Ö. F. Mapping global kimberlite potential from reconstructions of mantle flow over the past billion years. *PLOS ONE* **17**(6), 0268066. 10.1371/journal.pone.0268066 (2022). 10.1371/journal.pone.0268066 PMC9182341 35679269

[16] Zhang, Z. et al. Primordial metallic melt in the deep mantle. *Geophys. Res. Lett.* **43**(8), 3693–3699. 10.1002/2016GL068560 (2016).

[17] Gleeson, M., Soderman, C., Matthews, S., Cottaar, S. & Gibson, S. Geochemical constraints on the structure of the Earth’s deep mantle and the origin of the LLSVPs. *Geochem. Geophys. Geosyst.* **22**(9), 2021–009932. 10.1029/2021GC009932 (2021).

[18] Lee, C.-T.A. et al. Upside-down differentiation and generation of a ‘primordial’ lower mantle. *Nature* **463**(7283), 930–933. 10.1038/nature08824 (2010). 20164926 10.1038/nature08824

[19] Yuan, Q. et al. Moon-forming impactor as a source of Earth’s basal mantle anomalies. *Nature* **623**(7985), 95–99. 10.1038/s41586-023-06589-1 (2023). 37914947 10.1038/s41586-023-06589-1

[20] Tolstikhin, I. & Hofmann, A. W. Early crust on top of the Earth’s core. *Phys. Earth Planetary Interiors* **148**(2), 109–130. 10.1016/j.pepi.2004.05.011 (2005).

[21] Jones, T. D., Maguire, R. R., Van Keken, P. E., Ritsema, J. & Koelemeijer, P. Subducted oceanic crust as the origin of seismically slow lower-mantle structures. *Progress Earth Planetary Sci.* **7**, 17. 10.1186/s40645-020-00327-1 (2020).

[22] Niu, Y., Wilson, M., Humphreys, E. R. & O’Hara, M. J. A trace element perspective on the source of ocean island basalts (OIB) and fate of subducted ocean crust (SOC) and mantle lithosphere (SML). *Episodes J. Int. Geosci.* **35**(2), 310–327. 10.18814/epiiugs/2012/v35i2/002 (2012).

[23] Mulyukova, E., Steinberger, B., Dabrowski, M. & Sobolev, S. V. Survival of LLSVPs for billions of years in a vigorously convecting mantle: Replenishment and destruction of chemical anomaly. *J. Geophys. Res. Solid Earth* **120**(5), 3824–3847. 10.1002/2014JB011688 (2015).

[24] Panton, J., Davies, J. H. & Myhill, R. The stability of dense oceanic crust near the core-mantle boundary. *J. Geophys. Res. Solid Earth* **128**(2), 2022–025610. 10.1029/2022JB025610 (2023).

[25] Meer, D. G., Hinsbergen, D. J. J. & Spakman, W. Atlas of the underworld: Slab remnants in the mantle, their sinking history, and a new outlook on lower mantle viscosity. *Tectonophysics* **723**(2017), 309–448. 10.1016/j.tecto.2017.10.004 (2018).

[26] Davies, D. R., Goes, S. & Sambridge, M. On the relationship between volcanic hotspot locations, the reconstructed eruption sites of large igneous provinces and deep mantle seismic structure. *Earth Planetary Sci. Lett.* **411**, 121–130. 10.1016/j.epsl.2014.11.052 (2015).

[27] Sobolev, A. V. et al. The amount of recycled crust in sources of mantle-derived melts. *Science* **316**(5823), 412–417. 10.1126/science.1138113 (2007). 17395795

[28] Pietruszka, A. J., Norman, M. D., Garcia, M. O., Marske, J. P. & Burns, D. H. Chemical heterogeneity in the Hawaiian mantle plume from the alteration and dehydration of recycled oceanic crust. *Earth Planetary Sci. Lett.* **361**, 298–309. 10.1016/j.epsl.2012.10.030 (2013).

[29] Béguelin, P., Bizimis, M., Beier, C. & Turner, S. Rift-plume interaction reveals multiple generations of recycled oceanic crust in Azores lavas. *Geochimica et Cosmochimica Acta* **218**, 132–152. 10.1016/j.gca.2017.09.015 (2017).

[30] Cao, X., Flament, N. & Müller, R. D. Coupled evolution of plate tectonics and basal mantle structure. *Geochem. Geophys. Geosyst.* **22**(1), 2020–009244. 10.1029/2020GC009244 (2021).

[31] Li, Y., Deschamps, F. & Tackley, P. J. The stability and structure of primordial reservoirs in the lower mantle: Insights from models of thermochemical convection in three-dimensional spherical geometry. *Geophys. J. Int.* **199**(2), 914–930. 10.1093/gji/ggu295 (2014).

[32] Guerrero, J. M., Deschamps, F., Li, Y., Hsieh, W.-P. & Tackley, P. J. Influence of heterogeneous thermal conductivity on the long-term evolution of the lower-mantle thermochemical structure: implications for primordial reservoirs. *Solid Earth* **14**(2), 119–135. 10.5194/se-14-119-2023 (2023).

[33] Christensen, U. R. & Hofmann, A. W. Segregation of subducted oceanic crust in the convecting mantle. *J. Geophys. Res. Solid Earth* **99**(B10), 19867–19884. 10.1029/93JB03403 (1994).

[34] Huang, C., Leng, W. & Wu, Z. The continually stable subduction, iron-spin transition, and the formation of llsvps from subducted oceanic crust. *J. Geophys. Res. Solid Earth* **125**(1), 2019–018262. 10.1029/2019JB018262 (2020).

[35] Li, M. & McNamara, A. K. The difficulty for subducted oceanic crust to accumulate at the earth’s core-mantle boundary. *J. Geophys. Res. Solid Earth* **118**(4), 1807–1816. 10.1002/jgrb.50156 (2013).

[36] Baumgardner, J. R. Three-dimensional treatment of convective flow in the earth’s mantle. *J. Stat. Phys.* **39**(5–6), 501–511. 10.1007/BF01008348 (1985).

[37] Bunge, H.-P. & Baumgardner, J. R. Mantle convection modeling on parallel virtual machines. *Comput. Phys.* **9**(2), 207. 10.1063/1.168525 (1995).

[38] Müller, R. D. et al. A tectonic-rules-based mantle reference frame since 1 billion years ago—Implications for supercontinent cycles and plate-mantle system evolution. *Solid Earth* **13**(7), 1127–1159. 10.5194/se-13-1127-2022 (2022).

[39] Merdith, A. S. et al. Extending full-plate tectonic models into deep time: Linking the Neoproterozoic and the Phanerozoic. *Earth-Sci. Rev.* **214**, 103477. 10.1016/j.earscirev.2020.103477 (2021).

[40] Heck, H. J., Davies, J. H., Elliott, T. & Porcelli, D. Global-scale modelling of melting and isotopic evolution of Earth’s mantle: Melting modules for TERRA. *Geosci. Model Develop.* **9**(4), 1399–1411. 10.5194/gmd-9-1399-2016 (2016).

[41] Price, M. G., Davies, J. H. & Panton, J. Controls on the deep-water cycle within three-dimensional mantle convection models. *Geochem. Geophys. Geosyst.*[SPACE]10.1029/2018GC008158 (2019).

[42] Panton, J. et al. Investigating influences on the Pb pseudo-isochron using three-dimensional mantle convection models with a continental reservoir. *Geochem. Geophys. Geosyst.* **23**(8), 2021–010309. 10.1029/2021GC010309 (2022).

[43] Ritsema, J., Deuss, A., Van Heijst, H. J. & Woodhouse, J. H. S40RTS: A degree-40 shear-velocity model for the mantle from new Rayleigh wave dispersion, teleseismic traveltime and normal-mode splitting function measurements. *Geophys. J. Int.* **184**(3), 1223–1236. 10.1111/J.1365-246X.2010.04884.X (2011).

[44] Wang, W. et al. Velocity and density characteristics of subducted oceanic crust and the origin of lower-mantle heterogeneities. *Nat. Commun.* **11**(1), 1–8. 10.1038/s41467-019-13720-2 (2020). 31911578 10.1038/s41467-019-13720-2 PMC6946644

[45] Tsuchiya, T. Elasticity of subducted basaltic crust at the lower mantle pressures: Insights on the nature of deep mantle heterogeneity. *Phys. Earth Planetary Interiors* **188**(3–4), 142–149. 10.1016/J.PEPI.2011.06.018 (2011).

[46] Ricolleau, A. et al. Phase relations and equation of state of a natural MORB: Implications for the density profile of subducted oceanic crust in the Earth’s lower mantle. *J. Geophys. Res. Solid Earth* **115**(B8), 8202. 10.1029/2009JB006709 (2010).

[47] Rudolph, M.L., Lourenço, D.L., Moulik, P., & Lekić, V. Long-Wavelength Mantle Structure. In: Mantle Convection and Surface Expressions, pp. 1–19. American Geophysical Union (AGU), ??? (2021). 10.1002/9781119528609.ch1 . Accessed 2024-07-24

[48] Nomura, R. et al. Low core-mantle boundary temperature inferred from the solidus of pyrolite. *Science* **343**(6170), 522–525. 10.1126/science.1248186 (2014). 24436185 10.1126/science.1248186

[49] Alfè, D., Gillan, M. J. & Price, G. D. Temperature and composition of the Earth’s core. *Contemporary Phys.* **48**(2), 63–80. 10.1080/00107510701529653 (2007).

[50] Becker, T. W. & Boschi, L. A comparison of tomographic and geodynamic mantle models. *Geochem. Geophys. Geosyst.*[SPACE]10.1029/2001GC000168 (2002).

[51] Shephard, G. E., Matthews, K. J., Hosseini, K. & Domeier, M. On the consistency of seismically imaged lower mantle slabs. *Sci. Rep.* **7**(1), 10976. 10.1038/s41598-017-11039-w (2017). 28887461 10.1038/s41598-017-11039-w PMC5591187

[52] Hosseini, K. et al. SubMachine: Web-based tools for exploring seismic tomography and other models of Earth’s deep interior. *Geochem. Geophys. Geosyst.* **19**(5), 1464–1483. 10.1029/2018GC007431 (2018). 30174559 10.1029/2018GC007431 PMC6109961

[53] Yuan, Q. & Li, M. Instability of the African large low-shear-wave-velocity province due to its low intrinsic density. *Nat. Geosci.* **15**(4), 334–339. 10.1038/s41561-022-00908-3 (2022).

[54] Li, Y., Deschamps, F. & Tackley, P. J. Effects of the post-perovskite phase transition properties on the stability and structure of primordial reservoirs in the lower mantle of the Earth. *Earth Planetary Sci. Lett.* **432**, 1–12. 10.1016/j.epsl.2015.09.040 (2015).

[55] Li, M. Variable distribution of subducted oceanic crust beneath subduction regions of the lowermost mantle. *Phys. Earth Planetary Interiors* **341**, 107063. 10.1016/j.pepi.2023.107063 (2023).

[56] Doucet, L. S. et al. Distinct formation history for deep-mantle domains reflected in geochemical differences. *Nat. Geosci.* **13**(7), 511–515. 10.1038/s41561-020-0599-9 (2020).

[57] Hassan, R., Flament, N., Gurnis, M., Bower, D. J. & Müller, D. Provenance of plumes in global convection models. *Geochem. Geophys. Geosyst.* **16**(5), 1465–1489. 10.1002/2015GC005751 (2015).

[58] Jackson, M. G., Becker, T. W. & Steinberger, B. Spatial characteristics of recycled and primordial reservoirs in the deep mantle. *Geochem. Geophys. Geosyst.* **22**(3), 2020–009525. 10.1029/2020GC009525 (2021).

[59] Ritsema, J., McNamara, A. K. & Bull, A. L. Tomographic filtering of geodynamic models: Implications for models interpretation and large-scale mantle structure. *J. Geophys. Res. Solid Earth.*[SPACE]10.1029/2006JB004566 (2007).

[60] Ritsema, J., Van Heijst, H. J., Woodhouse, J. H. & Deuss, A. Long-period body wave traveltimes through the crust: Implication for crustal corrections and seismic tomography. *Geophys. J. Int.* **179**(2), 1255–1261. 10.1111/j.1365-246X.2009.04365.x (2009).

[61] Karato, S.-I. & Karki, B. B. Origin of lateral variation of seismic wave velocities and density in the deep mantle. *J. Geophys. Res. Solid Earth* **106**(B10), 21771–21783. 10.1029/2001JB000214 (2001).

[62] Jones, T. D., Sime, N. & Keken, P. E. Burying Earth’s primitive mantle in the slab graveyard. *Geochem. Geophys. Geosyst.* **22**(3), 2020–009396. 10.1029/2020GC009396 (2021).

[63] Gülcher, A. J. P., Ballmer, M. D. & Tackley, P. J. Coupled dynamics and evolution of primordial and recycled heterogeneity in Earth’s lower mantle. *Solid Earth* **12**(9), 2087–2107. 10.5194/se-12-2087-2021 (2021).

[64] Stixrude, L. & Lithgow-Bertelloni, C. Thermal expansivity, heat capacity and bulk modulus of the mantle. *Geophys. J. Int.* **228**(2), 1119–1149. 10.1093/gji/ggab394 (2022).

[65] Badro, J. et al. Iron partitioning in earth’s mantle: Toward a deep lower mantle discontinuity. *Science* **300**(5620), 789–791. 10.1126/science.1081311 (2003). 12677070 10.1126/science.1081311

[66] Ammann, M. W., Brodholt, J. P. & Dobson, D. P. Ferrous iron diffusion in ferro-periclase across the spin transition. *Earth Planetary Sci. Lett.* **302**(3), 393–402. 10.1016/j.epsl.2010.12.031 (2011).

[67] Trautner, V. E. et al. Compressibility of ferropericlase at high-temperature: Evidence for the iron spin crossover in seismic tomography. *Earth Planetary Sci. Lett.* **618**, 118296. 10.1016/j.epsl.2023.118296 (2023).

[68] Stixrude, L. & Lithgow-Bertelloni, C. Thermodynamics of mantle minerals—II. Phase equilibria. *Geophys. J. Int.* **184**(3), 1180–1213. 10.1111/j.1365-246X.2010.04890.x (2011).

[69] Bunge, H.-P., Richards, M. A. & Baumgardner, J. R. A sensitivity study of three-dimensional spherical mantle convection at 10 8 Rayleigh number: Effects of depth-dependent viscosity, heating mode, and an endothermic phase change. *J. Geophys. Res. Solid Earth* **102**(B6), 11991–12007. 10.1029/96JB03806 (1997).

[70] Bunge, H.-P., Hagelberg, C. R. & Travis, B. J. Mantle circulation models with variational data assimilation: Inferring past mantle flow and structure from plate motion histories and seismic tomography. *Geophys. J. Int.* **152**(2), 280–301. 10.1046/j.1365-246X.2003.01823.x (2003).

[71] McKenzie, D. P., Roberts, J. M. & Weiss, N. O. Convection in the earth’s mantle: Towards a numerical simulation. *J. Fluid Mech.* **62**(3), 465–538. 10.1017/S0022112074000784 (1974).

[72] Bunge, H.-P., Richards, M.A., & Baumgardner, J.R. Mantle-circulation models with sequential data assimilation: Inferring present-day mantle structure from plate-motion histories. *Philos. Trans. R. Soc. Lond. Series A Math. Phys. Eng. Sci*. **360**(1800), 2545–2567 (2002). 10.1098/rsta.2002.1080. 10.1098/rsta.2002.1080 12460480

[73] Crameri, F., Tackley, P. J., Meilick, I., Gerya, T. V. & Kaus, B. J. P. A free plate surface and weak oceanic crust produce single-sided subduction on Earth. *Geophys. Res. Lett.*[SPACE]10.1029/2011GL050046 (2012).

[74] Keken, P. E. & Ballentine, C. J. Whole-mantle versus layered mantle convection and the role of a high-viscosity lower mantle in terrestrial volatile evolution. *Earth Planetary Sci. Lett.* **156**(1–2), 19–32. 10.1016/s0012-821x(98)00023-5 (1998).

[75] Baker, M. B. & Beckett, J. R. The origin of abyssal peridotites: A reinterpretation of constraints based on primary bulk compositions. *Earth Planetary Sci. Lett.* **171**(1), 49–61. 10.1016/S0012-821X(99)00130-2 (1999).

[76] Walter, M.J. 2.08—Melt extraction and compositional variability in mantle lithosphere. in (Holland, H.D., Turekian, K.K., eds.) Treatise on Geochemistry, pp. 363–394. Pergamon, Oxford (2003). 10.1016/B0-08-043751-6/02008-9 . https://www.sciencedirect.com/science/article/pii/B0080437516020089 Accessed 2024-03-18

[77] White, W.M., & Klein, E.M. 4.13 - Composition of the Oceanic Crust. In: Holland, H.D., Turekian, K.K. (eds.) Treatise on Geochemistry (Second Edition), pp. 457–496. Elsevier, Oxford (2014). 10.1016/B978-0-08-095975-7.00315-6

[78] Wolstencroft, M. & Huw Davies, J. Breaking supercontinents; no need to choose between passive or active. *Solid Earth* **8**(4), 817–825. 10.5194/se-8-817-2017 (2017).

[79] Hirose, K., Takafuji, N., Sata, N. & Ohishi, Y. Phase transition and density of subducted MORB crust in the lower mantle. *Earth Planetary Sci. Lett.* **237**(1–2), 239–251. 10.1016/j.epsl.2005.06.035 (2005).

[80] Connolly, J. A. D. The geodynamic equation of state: What and how. *Geochem. Geophys. Geosyst.*[SPACE]10.1029/2009GC002540 (2009).

[81] Goes, S., Cammarano, F. & Hansen, U. Synthetic seismic signature of thermal mantle plumes. *Earth Planetary Sci. Lett.* **218**(3), 403–419. 10.1016/S0012-821X(03)00680-0 (2004).

[82] Maguire, R., Ritsema, J., Keken, P. E., Fichtner, A. & Goes, S. P- and S-wave delays caused by thermal plumes. *Geophys. J. Int.* **206**(2), 1169–1178. 10.1093/gji/ggw187 (2016).

[83] Xu, W., Lithgow-Bertelloni, C., Stixrude, L. & Ritsema, J. The effect of bulk composition and temperature on mantle seismic structure. *Earth Planetary Sci. Lett.* **275**(1–2), 70–79. 10.1016/j.epsl.2008.08.012 (2008).

[84] Davies, D. R. et al. Reconciling dynamic and seismic models of Earth’s lower mantle: The dominant role of thermal heterogeneity. *Earth Planetary Sci. Lett.* **353–354**, 253–269. 10.1016/j.epsl.2012.08.016 (2012).

[85] Peng, D. & Liu, L. Quantifying slab sinking rates using global geodynamic models with data-assimilation. *Earth-Sci. Rev.* **230**, 104039. 10.1016/j.earscirev.2022.104039 (2022).

[86] Pedregosa, F. et al. Scikit-learn: Machine learning in Python. *J. Machine Learn. Res.* **12**(85), 2825–2830 (2011).

[87] Crameri, F., Shephard, G. E. & Heron, P. J. The misuse of colour in science communication. *Nat. Commun.* **11**(1), 1–10. 10.1038/s41467-020-19160-7 (2020). 33116149 10.1038/s41467-020-19160-7 PMC7595127

