# Supplementary material for: Unique composition and evolutionary histories of large low velocity provinces
Source: Sci Rep. 2025 Feb 6;15:4466. doi: 10.1038/s41598-025-88931-3 (PMC11802776; doi:10.1038/s41598-025-88931-3)
Supplement: Supplementary file 1 — Supplementary Information. [file 41598_2025_88931_MOESM1_ESM.pdf]

Supplementary Information for: Unique composition and evolutionary histories of large low velocity provinces

James Panton[1*], J. Huw Davies[1], Paula Koelemeijer[2], Robert Myhill[3], Jeroen Ritsema[4]

[1*]School of Earth and Environmental Sciences, Cardiff University, Park Place, Cardiff, CF10 3AT, Wales, UK.
[2]Department of Earth Sciences, University of Oxford, South Parks Road, Oxford, OX1 3AN, England, UK.
[3]School of Earth Sciences, University of Bristol, Queens Road, Bristol, BS8 1RJ, England, UK.
[4]Department of Earth and Environmental Sciences, University of Michigan, 1100 North University Avenue, Ann Arbor, 40819, MI, USA.

*Corresponding author(s). E-mail(s): pantonjc@cardiff.ac.uk;

Content

- Supplementary Figures S1–S16
- Link to Supplementary Video SV1

Supplementary Figures

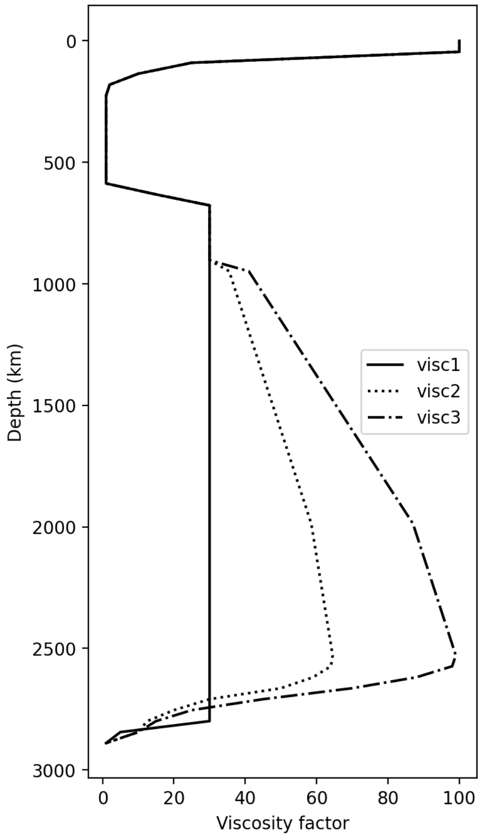

Fig. S1 Radial profile of the viscosity scaling factors used in the simulations listed in Table 1 of the main text.

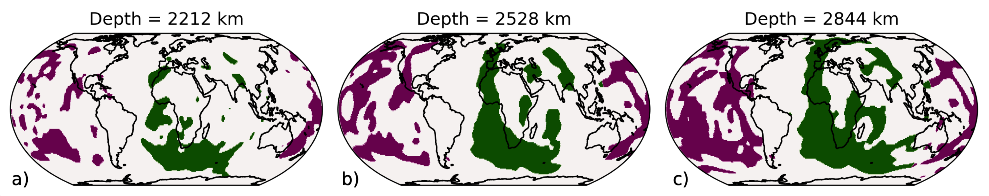

Fig. S2 Depth slices at 2212 km (a), 2528 km (b) and 2844 (km) to illustrate the identification of areas as s-LLVPs in the Pacific (purple) and African (green) regions for simulation RCY.

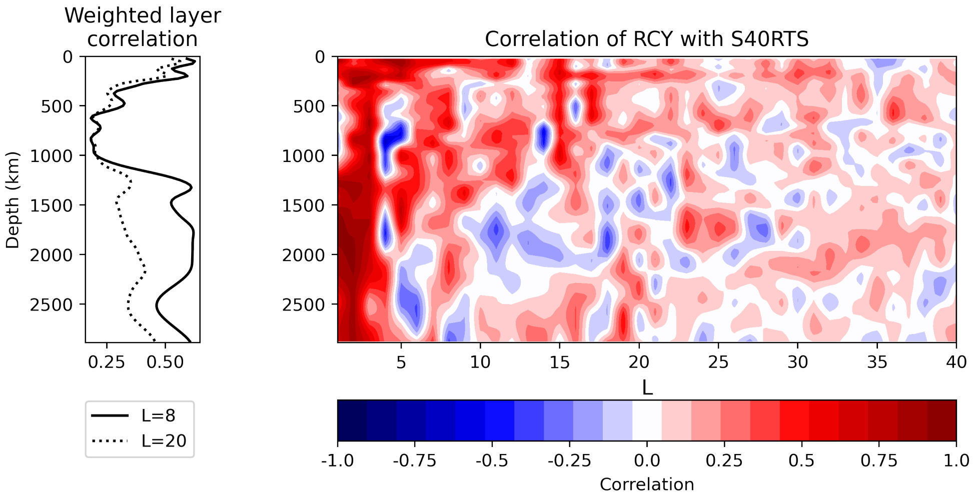

Fig. S3 Correlation between mantle structure predicted by simulation RCY and imaged by S40RTS versus depth and spherical harmonic degree, L (Methods 6.4), plotted up to $L = 40$ (right panel) or as a total weighted layer correlation up to $L = 8$ and $L = 20$ (left panel).

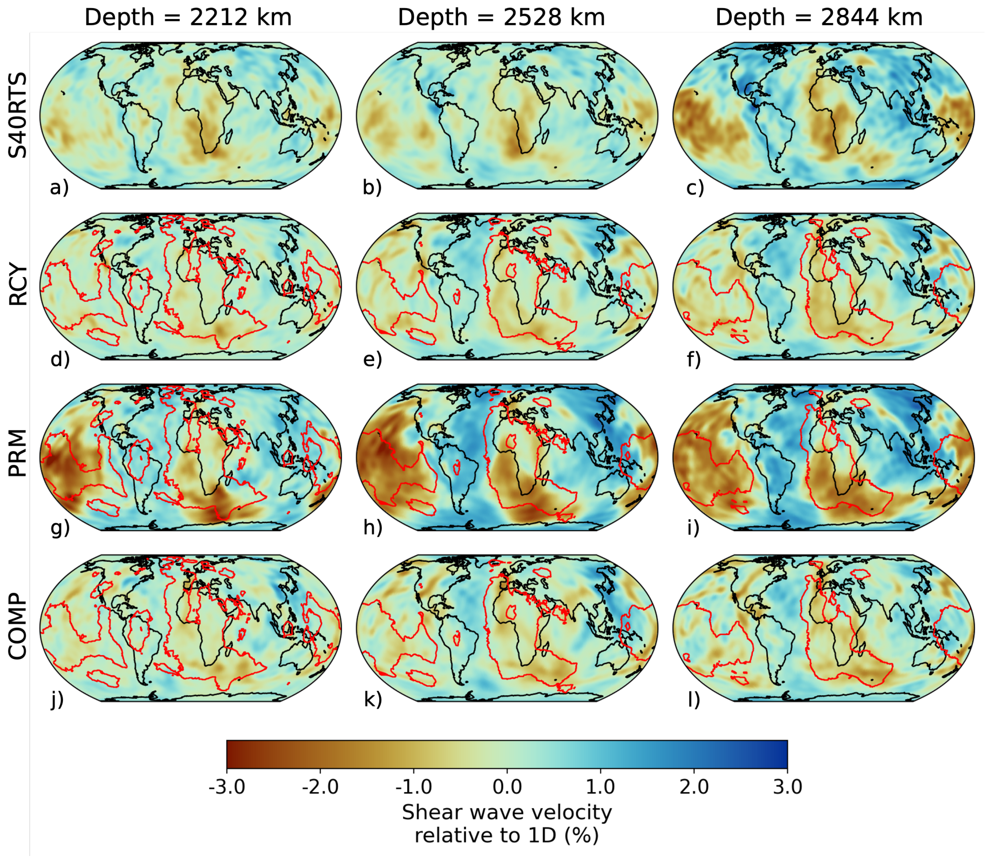

Fig. S4 Mantle structure at 2212 km (left column), 2528 km (centre column), and 2844 km (right column) depth, showing δV_s according to seismic tomography model S40RTS (a-c, top row), δV_s predicted in simulation RCY (d-f), δV_s predicted in simulation PRM (g-i) and δV_s predicted in simulation COMP (j-l), all filtered by the S40RTS resolution. Red outlines are derived from a vote map of low velocity regions detected in 18 shear wave tomography models [1, 2], indicating whereat least 5 models are in agreement.

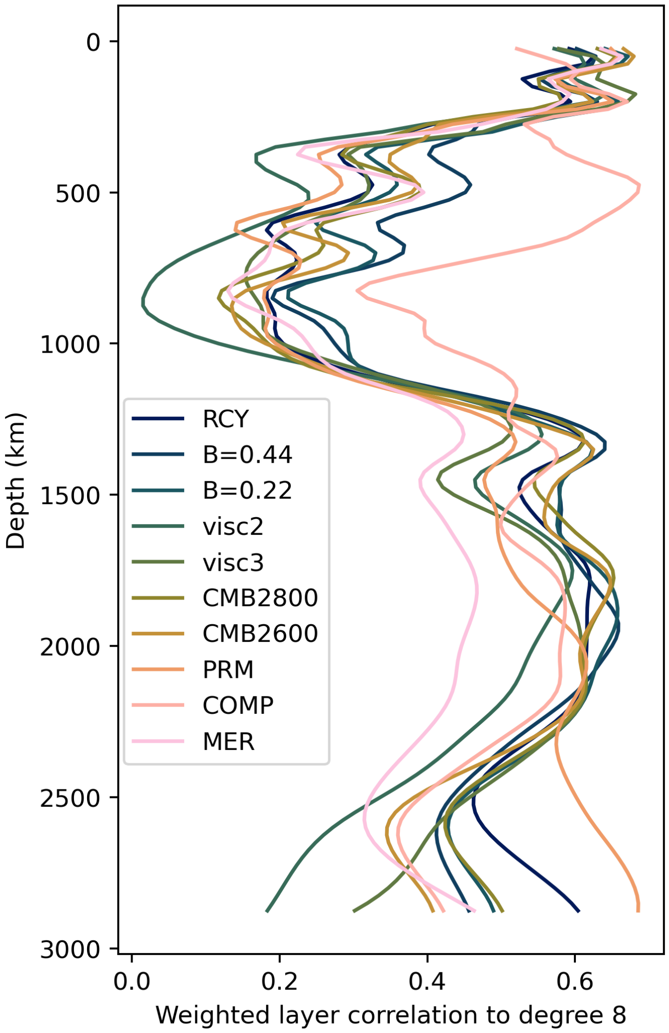

Fig. S5 Total weighted layer correlation (Methods 6.4) up to spherical harmonic degree $L = 8$ for all simulations.

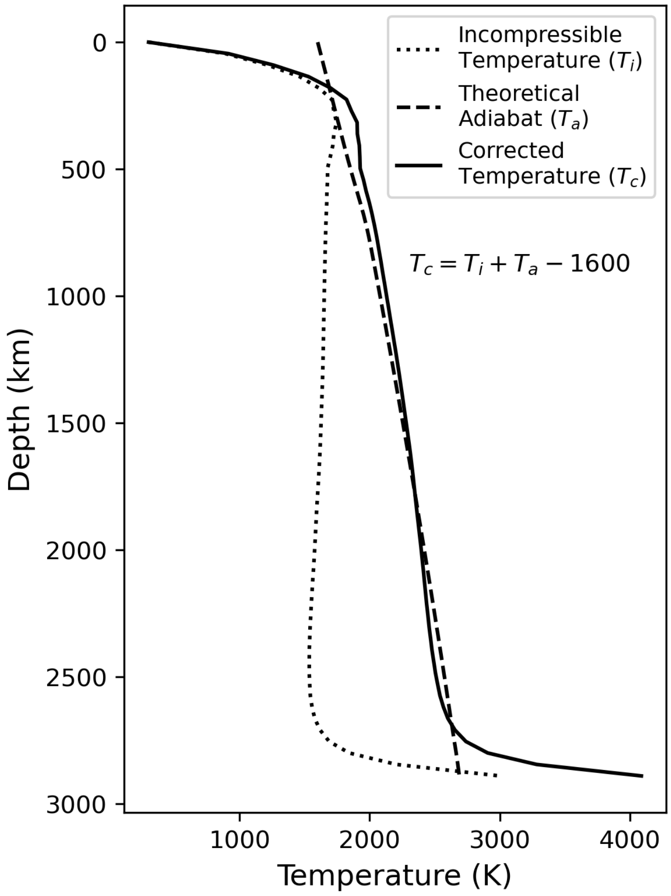

Fig. S6 Conversion of incompressible mantle temperature T_i to corrected temperature T_c using a theoretical adiabat.

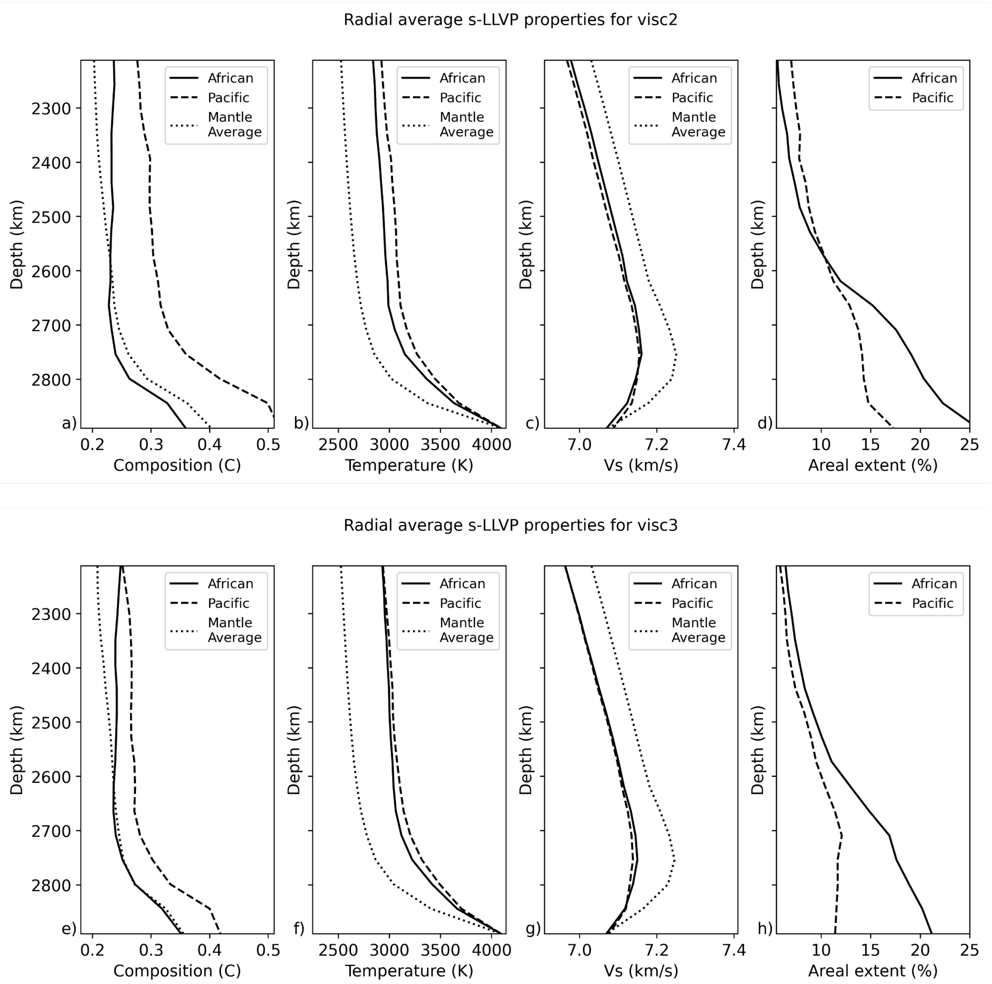

Fig. S7 Radial average of present day s-LLVP properties in simulations with different viscosity profiles: (a-d) visc2 and (e-h) visc3, showing (a,e) bulk composition, (b,f) temperature, (c,g) predicted absolute shear-wave velocity (unfiltered), and (d,h) areal extent in each radial layer, for both the African (solid line) and Pacific (dashed line) s-LLVPs identified in the simulations. Similar to Fig. S10 and Fig. 2 in the main text.

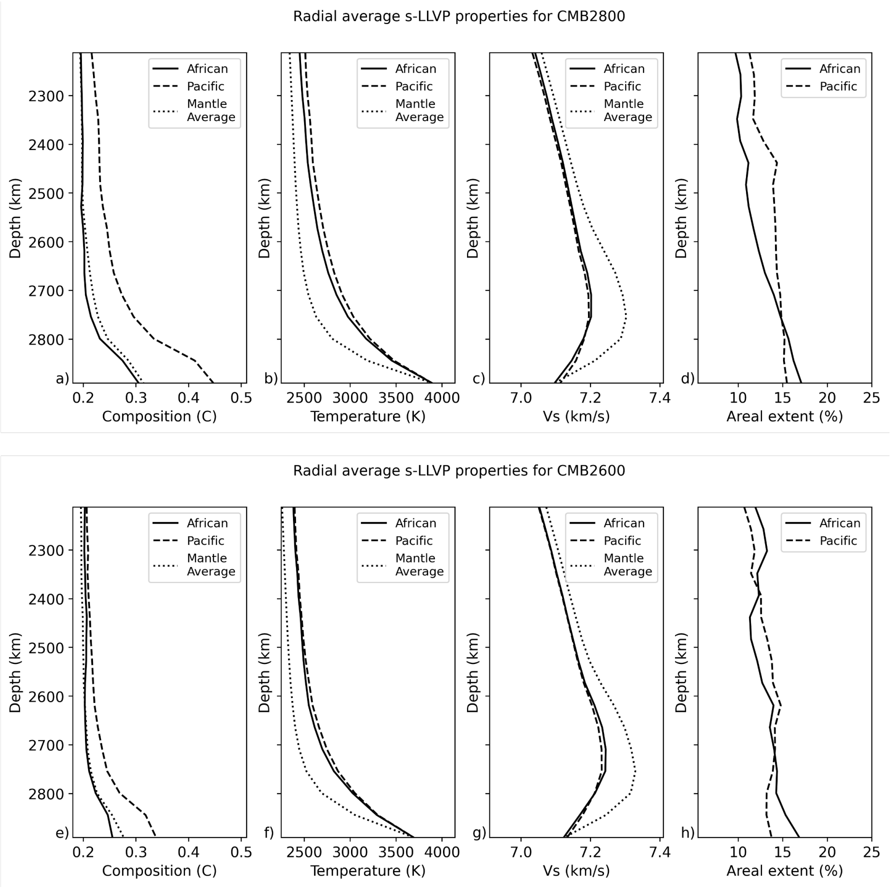

Fig. S8 Radial average of present day s-LLVP properties in simulations with different CMB temperatures: (a-d) CMB2800 and (e-h) CMB2600, showing (a,e) bulk composition, (b,f) temperature, (c,g) predicted absolute shear-wave velocity (unfiltered), and (d,h) areal extent in each radial layer, for both the African (solid line) and Pacific (dashed line) s-LLVPs identified in the simulations. Similar to Fig. S10-S7 and Fig. 2 in the main text.

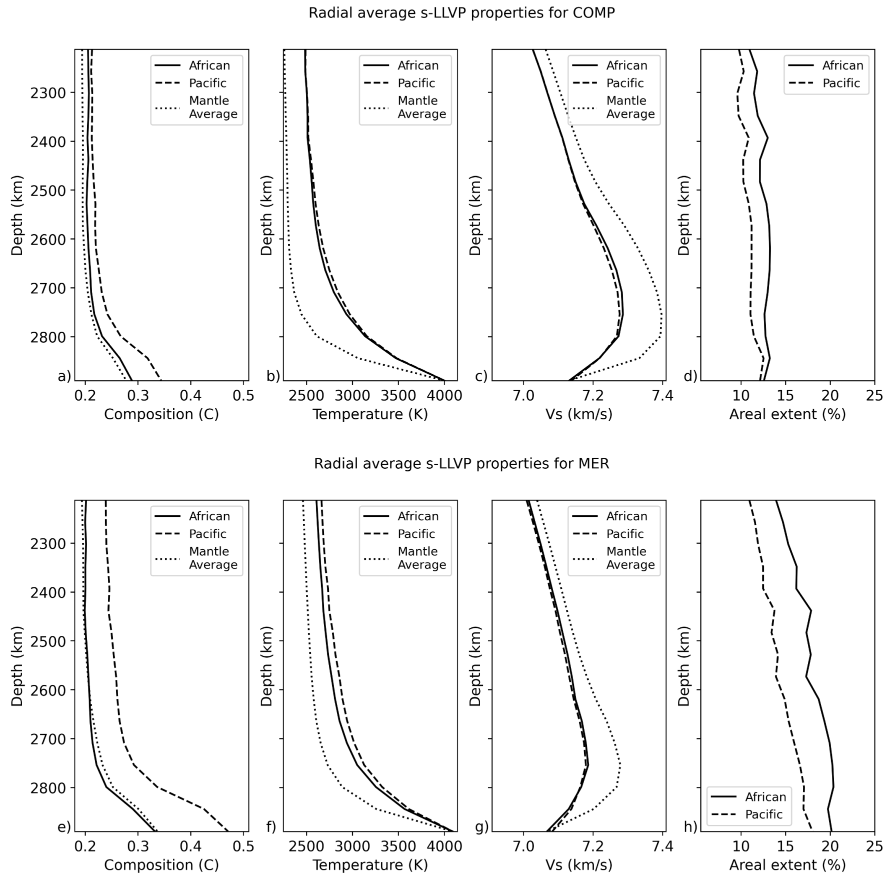

Fig. S9 Radial average of present day s-LLVP properties in simulations (a-d) COMP and (e-h) MER, showing (a,e) bulk composition, (b,f) temperature, (c,g) predicted absolute shear-wave velocity (unfiltered), and (d,h) areal extent in each radial layer, for both the African (solid line) and Pacific (dashed line) s-LLVPs identified in the simulations. Similar to Fig. S10-S8 and Fig. 2 in the main text.

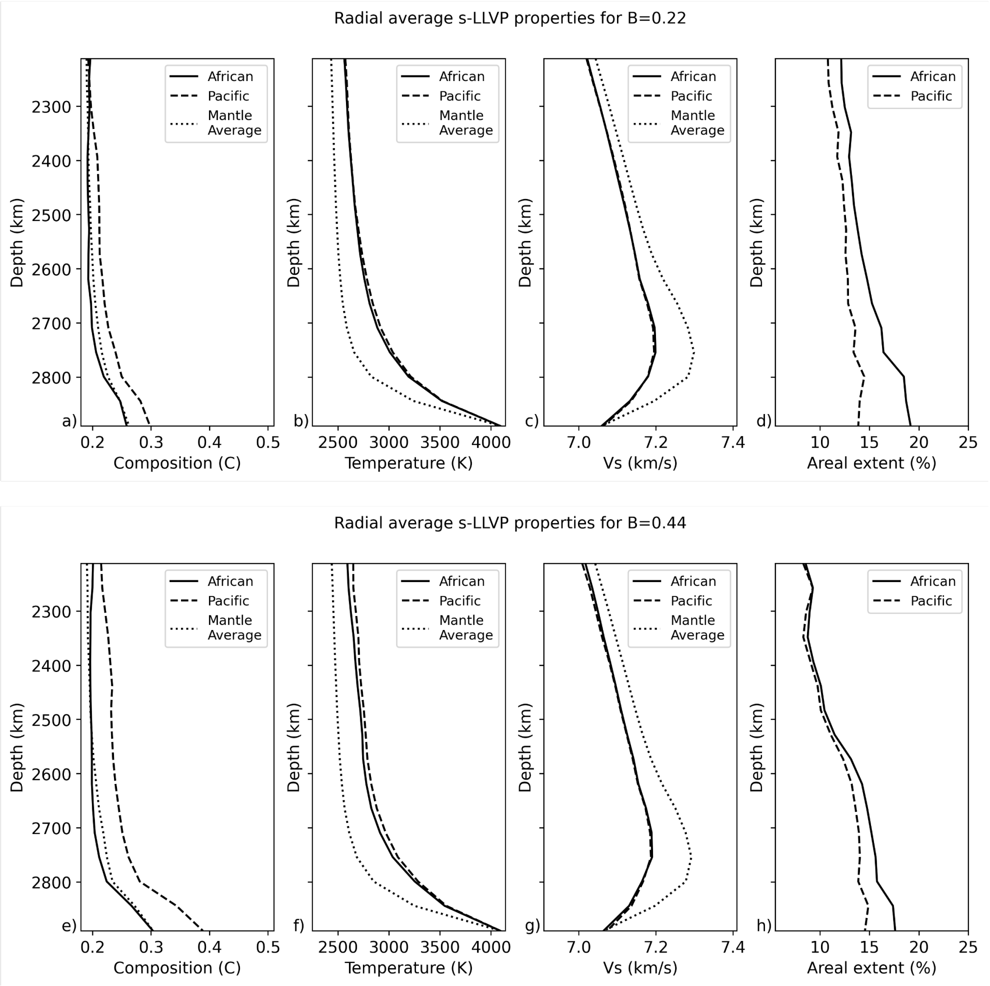

Fig. S10 Radial average of present day s-LLVP properties in simulations with different buoyancy numbers: (a-d) $B = 0.22$ and (e-h) $B = 0.44$, showing (a,e) bulk composition, (b,f) temperature, (c,g) predicted absolute shear-wave velocity (unfiltered), and (d,h) areal extent in each radial layer, for both the African (solid line) and Pacific (dashed line) s-LLVPs identified in the simulations. Similar to Fig. 2 in the main text.

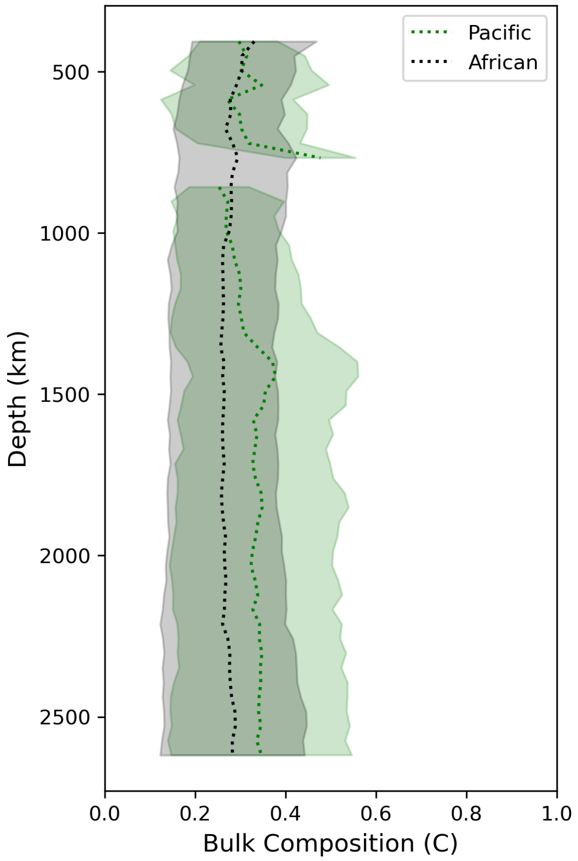

Fig. S11 Mean bulk composition of plumes associated with the Pacific and African s-LLVPs (dashed lines) in simulation RCY with ±1 standard deviation indicated by the shaded area.

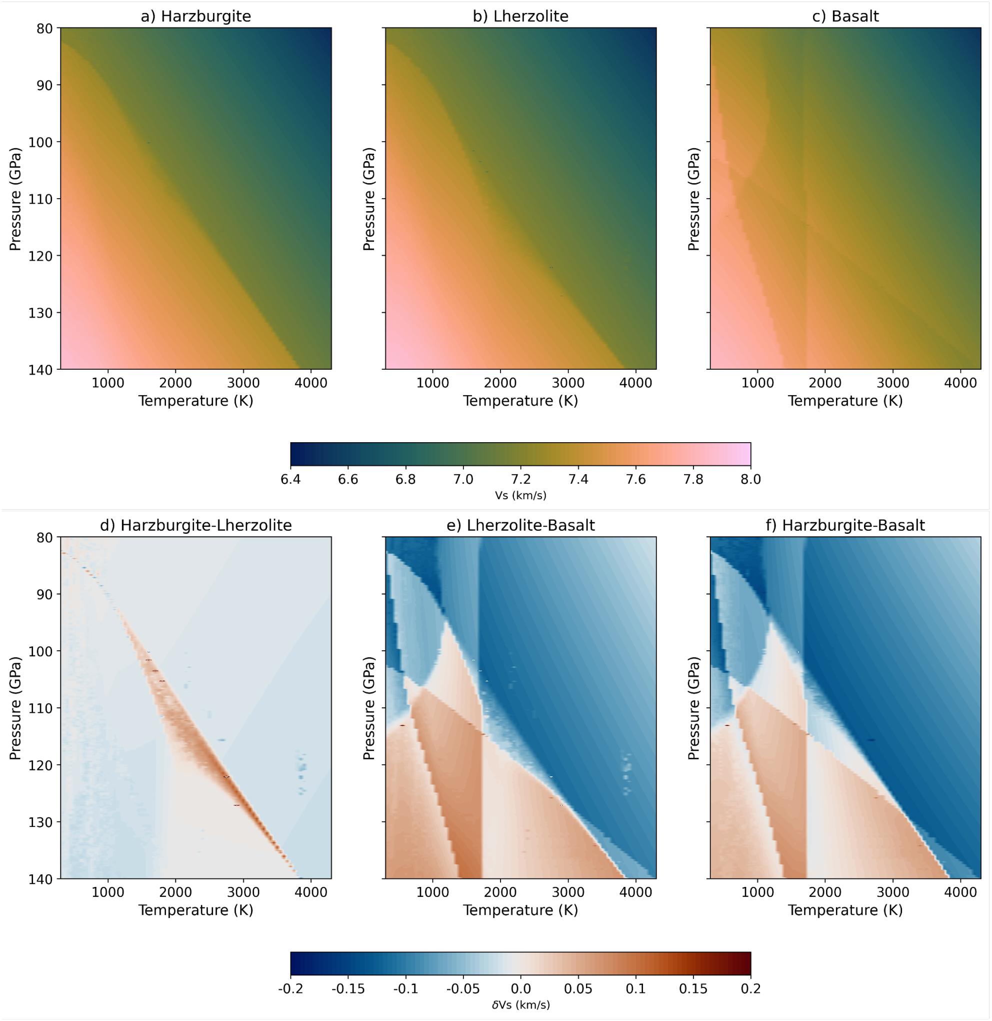

Fig. S12 Mineral physics estimates of the shear-wave velocity as a function of temperature and pressure. The top row show shows absolute V_s for the characteristic lithologies: (a) harzburgite, (b) lherzolite, and (c) basalt. The bottom row shows the differences in absolute V_s between (d) harzburgite and lherzolite, (e) lherzolite and basalt, and (f) harzburgite and basalt. See Methods 6.3 for full details of how the tables are produced.

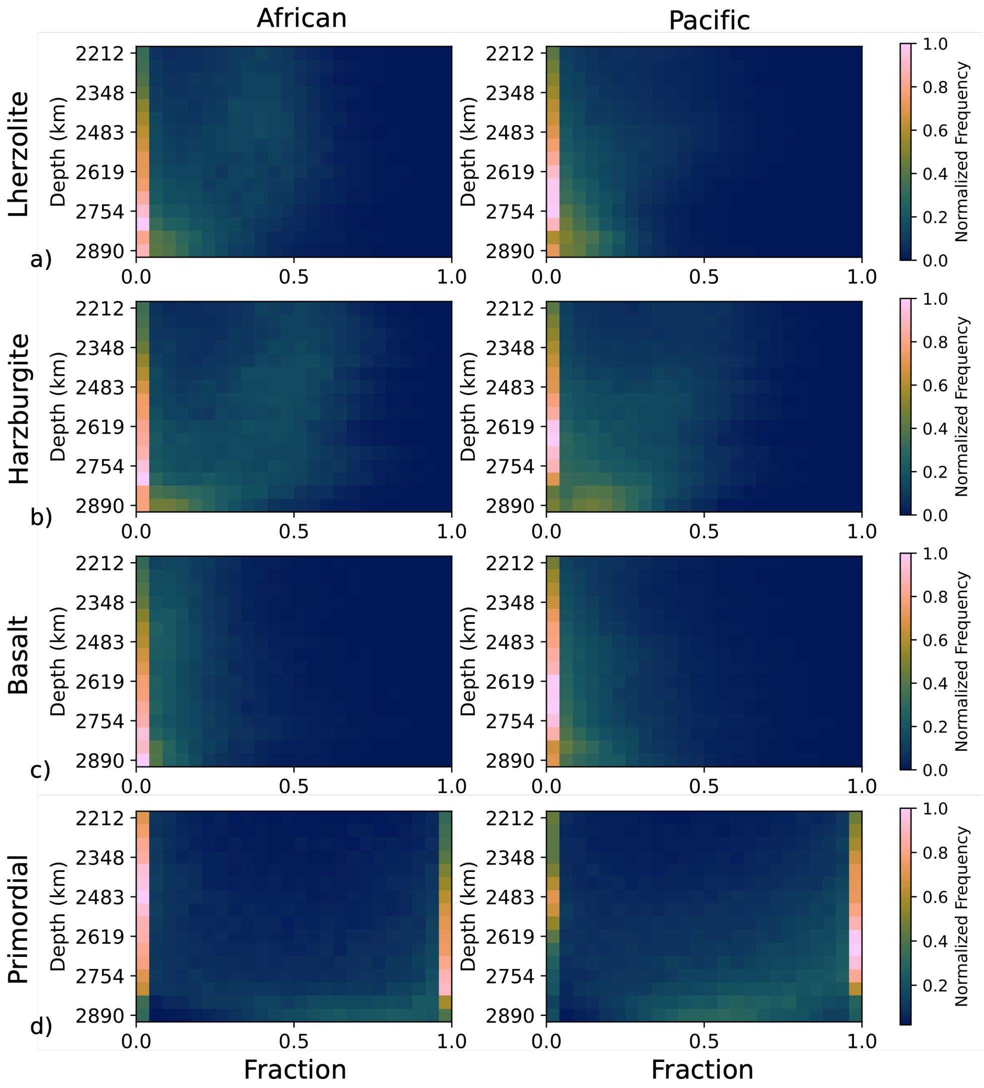

Fig. S13 Histograms for the proportions of different characteristic lithologies in the African (left column) and Pacific (right column) s-LLVPs in simulation PRM for each radial layer between 2212 km and 2890 km depth. The four characteristic lithologies are (a) harzburgite, (b) lherzolite, (c) basalt, and (d) the primordial component.

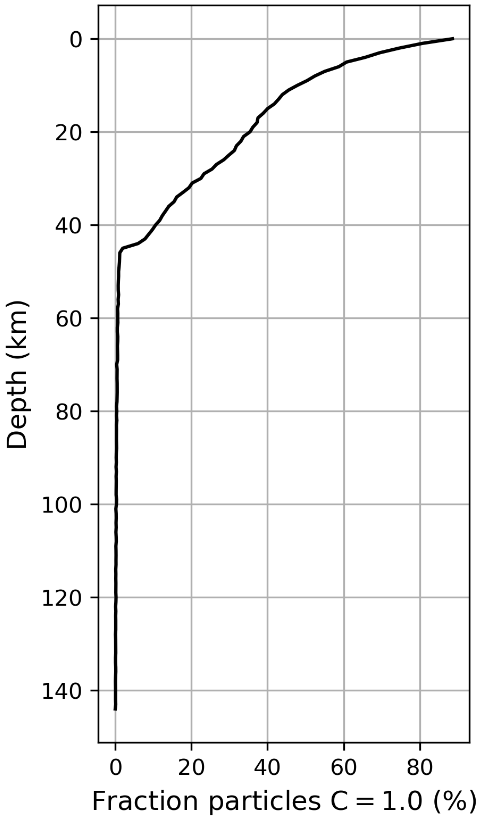

Fig. S14 Radial profile across the top 145 km of simulation RCY showing the fraction of particles within ocean basins that have a bulk composition of $C = 1.0$. The bottom of the oceanic crust is taken to be the depth at which 50% of particles in the ocean basins have a bulk composition of $C = 1$.

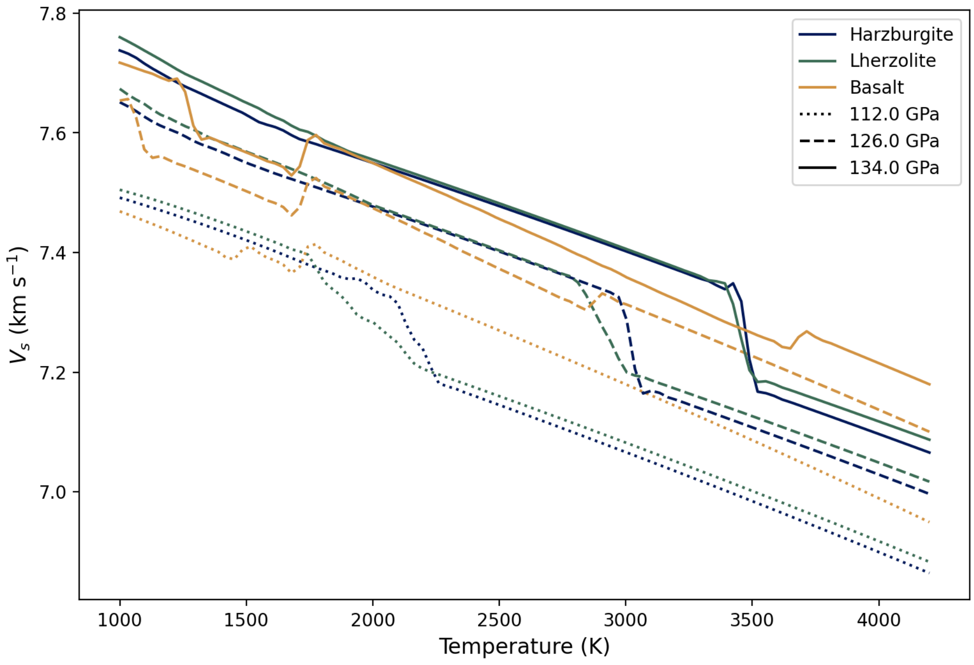

Fig. S15 Variation of predicted absolute V_s as a function of temperature at constant pressure, plotted for several characteristic lithologies: harzburgite (blue), lherzolite (green) and basalt (gold). Different lines indicate shear-wave velocities at different pressures: 134 GPa (solid line), 126 GPa (dashed line) and 112 GPa (dotted line).

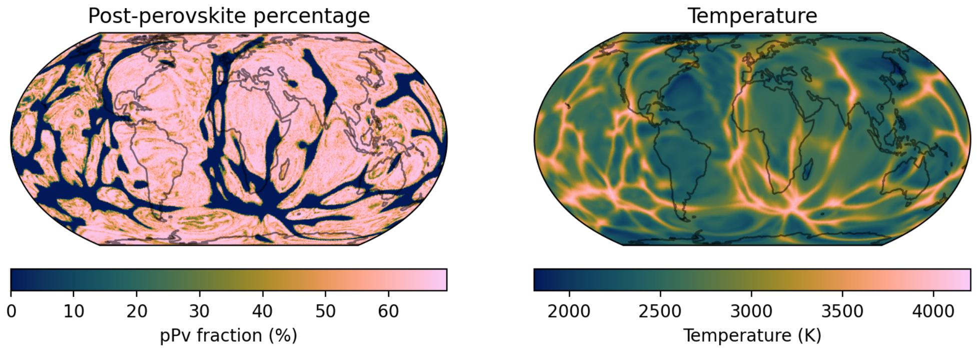

Fig. S16 Predicted distribution of post-perovskite in simulation RCY, shown at a depth of 2755 km. The left panel indicates the fraction of post-perovskite at this depth, the right panel shows the temperature field with an additional synthetic adiabat included.

Supplementary Video

Visualisation of case RCY.

Video SV1 https://youtu.be/Y_qnhtoa_dg

References

[1] Shephard, G.E., Matthews, K.J., Hosseini, K., Domeier, M.: On the consistency of seismically imaged lower mantle slabs. Scientific Reports **7**(1), 10976 (2017) https://doi.org/10.1038/s41598-017-11039-w . Publisher: Nature Publishing Group. Accessed 2024-07-25

[2] Hosseini, K., Matthews, K.J., Sigloch, K., Shephard, G.E., Domeier, M., Tsekhmistrenko, M.: SubMachine: Web-Based Tools for Exploring Seismic Tomography and Other Models of Earth's Deep Interior. Geochemistry, Geophysics, Geosystems **19**(5), 1464–1483 (2018) https://doi.org/10.1029/2018GC007431 . _eprint: https://onlinelibrary.wiley.com/doi/pdf/10.1029/2018GC007431. Accessed 2023-07-07
